# Studying the Impact of Different Field Environmental Conditions on Seed Quality of Quinoa: The Case of Three Different Years Changing Seed Nutritional Traits in Southern Europe

**DOI:** 10.3389/fpls.2021.649132

**Published:** 2021-05-12

**Authors:** Sara Granado-Rodríguez, Nieves Aparicio, Javier Matías, Luis Felipe Pérez-Romero, Isaac Maestro, Irene Gracés, Justo Javier Pedroche, Claudia Monika Haros, Nieves Fernandez-Garcia, Joaquín Navarro del Hierro, Diana Martin, Luis Bolaños, María Reguera

**Affiliations:** ^1^Department of Biology, Universidad Autónoma de Madrid, Madrid, Spain; ^2^Castile-Leon Agriculture Technology Institute (ITACyL), Valladolid, Spain; ^3^Agrarian Research Institute “La Orden-Valdesequera” of Extremadura (CICYTEX), Badajoz, Spain; ^4^Department of Agroforestry Sciences, Universidad de Huelva, Huelva, Spain; ^5^Department of Food & Health, Instituto de la Grasa, CSIC, Seville, Spain; ^6^Cereal Group, Institute of Agrochemistry and Food Technology (IATA-CSIC), Valencia, Spain; ^7^Department of Abiotic Stress and Plant Pathology, Centro de Edafología y Biología Aplicada del Segura (CSIC), Murcia, Spain; ^8^Departamento de Producción y Caracterización de Nuevos Alimentos, Instituto de Investigación enCiencias de la Alimentación (CIAL) (CSIC-UAM), Madrid, Spain; ^9^Sección Departamental de Ciencias de la Alimentación, Facultad de Ciencias, Universidad Autónoma de Madrid, Madrid, Spain

**Keywords:** quinoa, nutritional traits, seeds (grains), environmental adaptability, emerging crops, environment × genotype

## Abstract

*Chenopodium quinoa* Willd (quinoa) has acquired an increased agronomical and nutritional relevance due to the capacity of adaptation to different environments and the exceptional nutritional properties of their seeds. These include high mineral and protein contents, a balanced amino acid composition, an elevated antioxidant capacity related to the high phenol content, and the absence of gluten. Although it is known that these properties can be determined by the environment, limited efforts have been made to determine the exact changes occurring at a nutritional level under changing environmental conditions in this crop. To shed light on this, this study aimed at characterizing variations in nutritional-related parameters associated with the year of cultivation and different genotypes. Various nutritional and physiological traits were analyzed in seeds of different quinoa cultivars grown in the field during three consecutive years. We found differences among cultivars for most of the nutritional parameters analyzed. It was observed that the year of cultivation was a determinant factor in every parameter studied, being 2018 the year with lower yields, germination rates, and antioxidant capacity, but higher seed weights and seed protein contents. Overall, this work will greatly contribute to increase our knowledge of the impact of the environment and genotype on the nutritional properties of quinoa seeds, especially in areas that share climatic conditions to Southern Europe.

## Introduction

*Chenopodium quinoa* Willd, commonly known as quinoa, belongs to the Amaranthaceae family native to the Andean region (Wilson, 1990; Alandia et al., 2020). Its natural distribution is extended from northern Colombia to the southern region of Chile, and it can be cultivated in a wide range of altitudes, from sea level up to 4,000 m above sea level (Zurita-Silva et al., 2014). In the last decades, the cultivation of this crop has expanded worldwide, although the main producers in the world are still Bolivia and Peru (Bazile et al., 2016). One of the reasons for the increased interest in cultivating quinoa is the capacity of adaptation and its resilience to extreme conditions (Jacobsen et al., 2003). Quinoa can tolerate drought, high soil salinity, frost, and low temperatures (Jacobsen et al., 2005, 2012; Pulvento et al., 2010; Adolf et al., 2012), which makes it an ideal crop to be exploited and introduced in marginal environments (Choukr-Allah et al., 2016). On the other hand, the remarkable nutritional traits of quinoa seeds are key for its recent risen popularity for human consumption (Abugoch James, 2009). Quinoa is a very valued food for its high protein content, which is higher than that of cereal crops like barley, wheat, maize, and rice (Koziol, 1992). This protein is also of higher quality since it contains all amino acids essential for human consumption (including lysine, methionine, and cysteine). Furthermore, their proportions are well balanced and close to the amino acidic profile recommended by the Food and Agriculture Organization (FAO) (Filho et al., 2017). Quinoa seeds also contain fiber, vitamins, and minerals like calcium, zinc, magnesium, iron, potassium, phosphorus, manganese, copper, and sodium, and stand out for their unsaturated fatty acid contents and their large antioxidant capacity (Abugoch James, 2009; Paśko et al., 2009). The high content of vitamins (A, B, and E) and polyphenols, like phenolic acids or flavonoids, contribute to this high antioxidant capacity, and make of quinoa seeds an excellent example of “functional food,” since these antioxidants may prevent cancer, cardiovascular and other chronic diseases (Paśko et al., 2009; Tang and Tsao, 2017). Another interesting aspect of quinoa seed composition is their lack of gluten, which makes this food suitable for people with coeliac disease (Peñas et al., 2014). However, it should be noted that quinoa seeds also present important concentrations of antinutrient components such as saponins, which cause the characteristic bitter taste in non-desaponificated seeds (Ruales and Nair, 1993; Mastebroek et al., 2000). Nevertheless, saponins are also of current popularity as phytochemicals with bioactive properties for health (Marrelli et al., 2016; Singh et al., 2017; Navarro del Hierro et al., 2018; El Hazzam et al., 2020). Given quinoa’s nutritional quality and its ability to grow in a wide range of climatological conditions, the FAO considers that this crop has the potential for playing an important role in worldwide food security (Ruiz et al., 2014; FAO and CIRAD, 2015).

Given the high demand for quinoa, numerous breeding programs have been developed aiming to obtain new varieties better adapted to new agricultural areas. These breeding programs have been mainly focused on the generation of varieties less sensitive to the photoperiod, more resistant to downy mildew, with low saponin contents (sweet varieties), and with increased yields, all these aiming to satisfy the high demand for quinoa (Zurita-Silva et al., 2014). Grain size has also been used as a selection criterion in response to commercial appeal (Zurita-Silva et al., 2014). However, little attention has been paid to the improvement of nutritional properties in new varieties, even though food quality is as important as food quantity for food security (Tester and Langridge, 2010).

Nutritional quality in quinoa seeds is variable (Craine and Murphy, 2020), and this variability results from the interaction of genetic and environmental factors (Wimalasekera, 2015). Several studies have reported effects of the agrological conditions (Gonzalez et al., 2012; Prado et al., 2014; Präger et al., 2018; Reguera et al., 2018) and environmental factors such as water availability, soil salinity, climatic conditions, and temperature (Pulvento et al., 2012; Miranda et al., 2013; Bascuñán-Godoy et al., 2015; Aloisi et al., 2016; Lesjak and Calderini, 2017; Curti et al., 2018) on nutritional traits of quinoa seeds. However, there is still limited knowledge about the mechanisms that trigger these changes in nutrient content and whether the seed nutritional traits are stable depending on the genotype, environment, and GXE interaction.

Thus, the aim of this study was to identify and evaluate changes in the nutritional properties of quinoa seeds linked to the genotype and those related to changes in the environmental conditions. For this purpose, six bred cultivars were grown during three consecutive years (2017, 2018, and 2019) and different physiological and nutritional parameters were determined. The results presented here suggest that environmental factors heavily influenced most of the nutritional parameters analyzed.

## Materials and Methods

### Plant Material, Experimental Design, and Location

Quinoa cultivars Regalona (registered variety of BAER, Chile), Puno, Titicaca, and Vikinga (Quinoa Quality, Denmark), Q3 and Q5 (International Center for Biosaline Agriculture (ICBA), Dubai, United Arab Emirates) were cultivated in the Experimental Station of Zamadueñas, which belongs to the Instituto Tecnológico Agrario de Castilla y León (ITACyL), in Valladolid (Spain) (41°42′N and 4°42′W, 690 m.a.n.l) under irrigation in three consecutive growing seasons (2017, 2018, and 2019).

Climatological data (as a monthly average of daily data), including total precipitation and total irrigation, is presented in Supplementary Table 1. The data was obtained from a local climatological station located at the field experimental station of Zamadueñas. In 2017 and 2019, quinoa plants were sown in the spring (April and May, respectively). In 2018, due to heavy precipitations, the sowing date was delayed until June 18th.

The soil, containing 42% silt, 17% clay, and 41% sand, was a clay-silty-loam type presenting a pH ranging from 8.2 to 8.55. It contained 2.03–1.19% organic matter, and 0.053–0.083 dS.m^–1^ of electrical conductivity (EC) of the saturated paste. Phosphorous content (as ppm of P_2_O_5_) ranged from 48 to105, potassium content (as ppm of K_2_O) ranged from 0.33 to 0.472 and total nitrogen (%) ranged from 0.067 to 0.075 (Supplementary Table 2). The basal fertilization in the plots consisted of 300 kg ha^–1^ of 8-15-15 (NPK) in addition to top dressing of 500 kg ha^–1^ of NSA (26%) at six-leaves stage. The trials were kept free of weeds and pests.

The experimental design consisted of randomized blocks with 3 replications. Each block was 8.0 m long and 3.0 m broad (24 m^2^) with 6 rows separated 0.50 m. Sowing was carried out mechanically using a sowing density of 10 kg/ha between 1 cm and 2 cm depth. Harvesting was also carried out mechanically.

### Seed Weight and Seed Area

Seeds were manually counted and weighed in an analytical balance. Seed area was analyzed using the open-source software ImageJ^1^. Images were taken using an Olympus SZ61 stereomicroscope (Olympus Corporation, Shinjuku, Tokyo, Japan) and processed with the AnalySIS GetIT image software (analysis getIT 5.1, Olympus Corporation).

### Color

Color parameters were determined as described by Guiotto et al. (2020).

### Seed Germination Rate

Quinoa seeds were sterilized first in ethanol 70% (2 min), followed by a wash in bleach 50% with a droplet of Tween-20 (2 min) and then rinsed several times in distilled water (H20). Sterilized seeds were sown on a double layer of paper filter wet with distilled water on Petri dishes and then transferred to a growth chamber under darkness and a controlled temperature of 23°C. Germinated seeds (considered as germinated when the radicle protrusion was longer than 2 mm) were counted daily for the first week after sowing.

### Seed Viability

Seed viability was performed using the tetrazolium method (2,3,5-triphenyl-2*H*-tetrazolium chloride). First, seeds were imbibed in distilled water at 30°C for an hour in order to facilitate longitudinal and superficial cuts of the embryo and to ensure a homogeneous dying of the seed tissues. After cutting, seeds were submerged in 1% tetrazolium chloride at 30°C for 2 h. Seeds with more than 50% staining in the embryonic tissue were considered viable.

### Saponin Content

Saponins were quantified following the protocol described by Navarro del Hierro et al. (2020) based on a previous extraction with methanol assisted by ultrasound and a subsequent analysis by HPLC-DAD.

### Protein Content

The protein content was determined according to AOAC Official Methods (AOAC, 2000), using an elemental analyzer (Leco TruSpec) and considering a conversion factor of 6.25 (Nascimento et al., 2014).

### Amino Acid Quantification

Amino acid analysis was performed following the protocol described by Villanueva et al. (1999).

### Mineral Content

The mineral content was analyzed following the official methods of analysis of the Spanish Ministry of Agriculture (MAPA, 1995). The phosphorus content was determined using a spectrophotometer UV-VIS (Hitachi U-2810) (yellow coloration, 430 nm). Potassium was determined using flame atomic emission spectroscopy. Calcium, magnesium, sodium, copper, manganese, zinc, and iron content were assessed using flame atomic absorption spectroscopy (AAS) (SpectrAA 110, Agilent) after mineralizing the samples with H20 and HCl (35%).

### Evaluation of Antioxidant Capacity: Ferric Reducing Antioxidant Power (FRAP) Assay, Total Phenolic Content (TPC), and Total Flavonoid Content (TFC)

Total extracts were obtained from 100 mg of ground seeds, that were homogenized in 1 ml of an extraction buffer consisting of methanol (50%), acetic acid (1%), and distilled water (49%). The samples were then vortexed for 2 min and centrifuged for 15 min at 10,000 rpm. The supernatants were stored at −20°C until their use in the FRAP, phenol, and flavonoid assays.

#### Ferric Reducing Antioxidant Power (FRAP) Assay

The antioxidant capacity of seed samples was determined following the procedure described by Benzie and Strain (1996). The FRAP reagent consisted of a mix of 300 mM acetate buffer (pH 3.6), with 10 mM TPTZ in 40 mM HCl and 20 mM FeCl_3_⋅6H_2_O at a ratio of 10:1:1 (v/v/v). Twenty μl of sample extract and 180 μl of FRAP reagent were added into a 96-well microplate and, after 4 min, absorbance was read at 593 nm using a microplate reader (Lector Multi-ModalSynergy HTX, BioTek Instruments, Inc., United States). The antioxidant capacity was calculated from a calibration curve obtained with iron (II) sulfate (FeSO_4_). FRAP value was expressed as μmol of Fe^2+^/g of seed.

#### Total Phenol Content (TPC)

The content of polyphenols was measured following the protocol described by Tang et al. (2015). Briefly, the mixture of 50 μl of sample extract, 50 μl of the Folin-Ciocalteu reagent 10%, and 100 μl of sodium carbonate 13% was incubated for 60 min. Absorbance was read at 750 nm using a microplate reader (Lector Multi-ModalSynergy HTX, BioTek Instruments, Inc., United States). The TPC was expressed as mg of gallic acid equivalents per g of quinoa seed (mg GAE/g).

#### Total Flavonoid Content (TFC)

Flavonoid content was determined following the procedure described by Valenzuela-Bustamante (2015). Briefly, 30 μl of sample extract, 10 μl of aluminum chloride (AlCl_3_) 10%, 10 μl of sodium acetate (NaC_2_H_3_O_2_) 1M, and 250 μl of dH_2_O were mixed and incubated for 30 min. The absorbance was read at 415 nm using a microplate reader (Lector Multi-ModalSynergy HTX, BioTek Instruments, Inc., United States). The results were expressed in mg of quercetin equivalents per g of quinoa seed (mg QE/g).

### Statistical Analysis

To analyze the Genotype x Year interaction, two-way ANOVA was performed. Normality and equality of variances of the data were tested through a Kolmogorov-Smirnov’s test and a Levene’s, respectively. For variables where normality and equal variances could be assumed, a One-way ANOVA test was performed, followed by a Tukey post-hoc test, to perform multiple comparisons at a probability level of 5% (*p* < 0.05). A Krustal-Wallis test by ranks was performed when data did not present a normal distribution and a Welch’s ANOVA test followed by a Games-Howell post-hoc test was performed when variances were not equal, both at a probability level of 5% (*p* < 0.05).

Correlations amongst variables were evaluated with a Pearson’s correlation coefficient test and simple linear regressions were performed to analyze the relation between yield and qualitative seed variables. A sequential path analysis was performed to evaluate the specific contribution of nutritional seed traits to germination rate. This analysis allows ordering different variables as predictors of seed germination rate of first, second, or third-order (Mohammadi et al., 2003). For this purpose, a stepwise multiple linear regression procedure was used where variables that showed weak contribution (*p* > 0.05) to the dependent variable (germination rate) or high multicollinearity, were automatically dropped from the model. The variables entered into the model were considered as first-order predictors and the procedure was repeated using these variables as the response variable to identify traits that function as second-order predictors of germination rate. Tolerance and variance inflation factor (VIF) were used to measure the level of multicollinearity for each predictor trait, considering tolerance lower than 0.1 or VIF values higher than 10 as high levels of collinearity. Tolerance (1- *R^2^_*i*_*, where *R^2^_*i*_* is the coefficient of determination for the prediction of variable *i* by the predictor variables) is the amount of variance of the selected independent variable not explained by other independent variables. VIF (1/Tolerance) indicates the extent of effects of other independent variables on the variability of the selected independent variable. Principal component analysis was performed for viability and germination rates, yield, 1000 seeds’ weight, seed area, all color parameters, protein content, amino acid contents, FRAP value, phenols and flavonoids contents, and mineral contents. The SPSS Statistics 23.0 (SPSS Inc.) package was used for the statistical analyses.

## Results

### Plant Performance During the Three Consecutive Years

**FIGURE 1:**
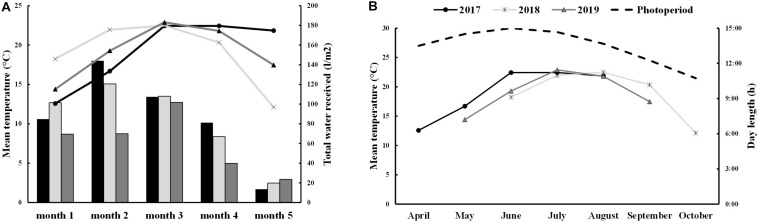
Environmental conditions **(A)** Total water supply and mean temperature for each month of development. The 2017 data is shown in black bars and lines, 2018 data in light gray bars and lines and 2019 data in dark gray bars and lines. **(B)** Mean temperatures in 2017 (black line), 2018 (light gray line), and 2019 (dark gray line) and day-length (dashed black line) in each month.

Field trials were performed in three consecutive growing seasons (2017, 2018, and 2019) using 6 different cultivars: Titicaca, Vikinga, Regalona, Puno, Q3, and Q5. In seasons 2017 and 2019, quinoa seeds were sown in April and May, respectively. However, in 2018, due to heavy rainfalls during the spring, sowing was delayed till June, which explains why higher minimum temperatures (accompanied with higher precipitations) were registered during the first month of cultivation (Supplementary Table 1 and Figure 1). Quinoa life cycle spans about 5 months, therefore, plants that were sown in 2017 and 2019 were harvested in the summer (August 31st and September 20th, respectively), facing higher maximum temperatures during their last months of growth, which include grain maturation (Supplementary Table 1). On the contrary, plants cultivated in 2018 were exposed to lower temperatures (5–10°C) in the last month before harvesting on October 25th (Supplementary Table 1). Furthermore, differences appeared among varieties when evaluating their lifespan (Supplementary Figure 1). Thus, while Puno cv., Titicaca cv., Q5 cv., and Regalona cv. showed similar phenological stages, Q3 cv. and Vikinga cv. presented longer life cycles.

Precipitations along the different growing seasons were very variable. In 2019, precipitations were low the first two months of cultivation (6.0–7.0 mm) increasing during the last three months. In 2017, the range of precipitations among months was wider, with rainfalls concentrated during the second and fourth months of cultivation (May and July). The second growing season, 2018, presented more extreme conditions with very high precipitations during the first month of cultivation (June, 70.6 mm) and no rainfall in the third month (August) (Supplementary Table 1 and Figure 1). Nonetheless, the irrigated conditions minimized the differences caused by the low precipitations in terms of water supply, being 2019 the year that showed a reduced water supply especially during the first two months of growth.

Total seed yield ranged from 0.70 t/ha to 3.25 t/ha, being Vikinga cv. 2019 an outlier, with a seed yield of 0,23 t/ha (Supplementary Figure 1). There were important yield differences among years: 2017 was the growing season that presented the highest yields (from 2.2 t/ha the Q3 cultivar to 3.25 t/ha Puno cv.). In contrast, 2018 showed the lowest yields except for Puno cv. and Q3 cv., which produced almost three times as much seed as the other cultivars.

**FIGURE 2:**
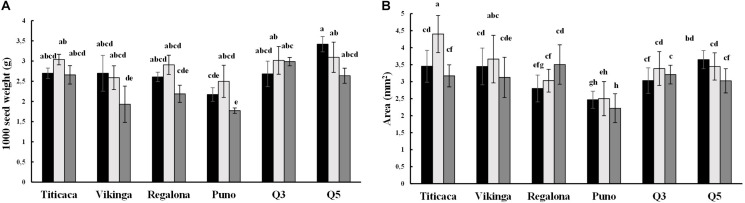
Seed weight and area. Black bars represent 2017 values, light gray bars show 2018 values, and 2019 values are represented by dark gray bars. **(A)** Thousand seed weight An ANOVA test followed by a post-hoc test Tukey was performed. **(B)** Seed area. A Welch’s ANOVA test followed by a Games-Howell post-hoc test was performed. Bars that do not share the same letters show statistically significant differences.

Seed area and seed weight showed an effect related to the cultivar, year of sowing, and cultivar x year interaction (*p* < 0.05) (Figure 2 and Supplementary Table 3). One thousand seeds’ weight ranged from 1.7 to 3.4 g and was significantly higher in seeds harvested in 2018 and 2017 compared to 2019 seeds (Figure 2A). Seeds from the 2018 harvest were also larger compared to the seeds from other years, while seeds from 2019 presented the smallest areas (Figure 2B). When comparing cultivars, Titicaca cv. and Q5 cv. seeds were both heavier and larger while Puno cv. seeds were the smallest and lightest. Interestingly, Vikinga cv. seeds showed lower weights than those from the Q3 cultivar, but larger areas. Q5 cv. 2017 seeds presented the highest seed weight, followed by Titicaca cv. 2018. Both also showed the largest areas, being Titicaca cv. 2018 seeds twice as wide as the lightest and smallest seeds harvested from Puno cv. in 2019 (4.4 and 2.2 mm^2^, respectively). No correlation between these two parameters was found (Supplementary Figure 4), which indicates that seeds may differ in density and shapes depending on the cultivar and the environmental conditions.

### Germination Rates and Seed Viability

**FIGURE 3:**
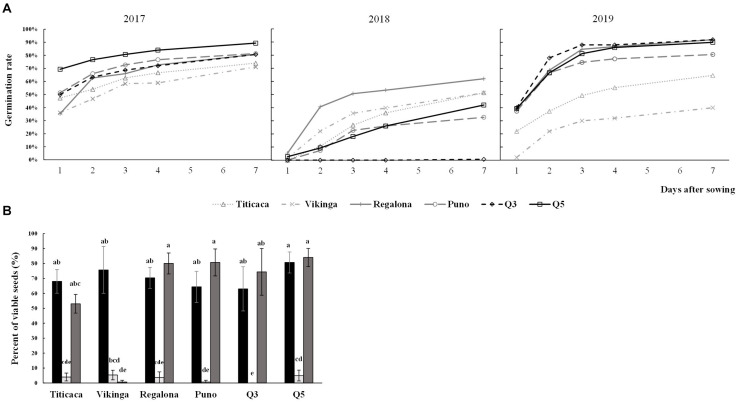
Germination rates and seed viability. **(A)** Germination rates. Evolving germination rate is shown over the 7 first days after sowing seeds harvested in three consecutive years. **(B)** Seeds viability was evaluated using the tetrazolium method. Black bars, light gray bars, and dark gray bars indicate 2017 seeds, 2018 seeds, and 2019 seeds, respectively. A Welch’s ANOVA test followed by a Games-Howell *post-hoc* test was used to compare the data of germination rate at seven days after sowing, and a Krustal-Wallis test by ranks was performed for viability rate. Bars that do not share letters are significantly different.

To evaluate the germination capacity of the seeds, germination rates were determined for all cultivars harvested in the three consecutive years (Figure 3). The year of cultivation, the cultivar, and the interaction of these two factors had a significant influence on the germination rates (*p* < 0.05) (Supplementary Table 3). The results showed that 2018 was the year in which seeds showed lower germination rates, being Regalona the cultivar with the highest rate and Q3 the only variety that did not germinate. Most of the varieties showed similar germination trends in 2017 and 2019, except for Vikinga cv. and Titicaca cv. In 2019, these two cultivars showed similar rates to those obtained for the 2018 growing season.

Seed viability also presented high influence of year, cultivar, and the year x cultivar interaction, and was well correlated with the germination data (Supplementary Figure 4). Thus, 2018 seeds showed a steeped decrease in seed viability and Vikinga cv. seeds’ viability from the 2019 harvest was hardly measurable (Figure 3B).

### Saponin Content

**FIGURE 4:**
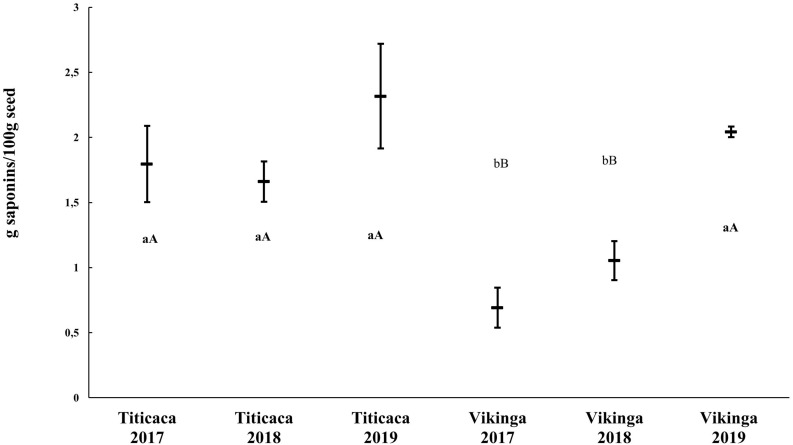
Saponin content in quinoa seeds. Saponin content of Titicaca cv. and Vikinga cv. seeds sown and harvested in different years is presented. Different lower-case letters indicate significant differences between cultivars within a year and upper-case letter show differences between years within a cultivar. *T-student* comparisons were performed between pairs of samples.

Saponins are secondary metabolites whose content may vary when changing the environmental conditions (Szakiel et al., 2011). To analyze the effects of environmental conditions on saponin content in quinoa seeds, we studied the saponin content of two different cultivars: Titicaca cv., considered a bitter cultivar due to the higher concentration of saponin in its seeds, and Vikinga cv., a sweet cultivar with lower saponin content. Indeed, significant differences were found between the two cultivars, Titicaca cv. seeds containing more saponins than Vikinga cv. seeds (an average of 1.92 g/100 g and 1.26 g/100 g, respectively). Furthermore, there was an important increase in saponins in 2019, especially in Vikinga cv. seeds (2.04 g/100 g), that increased the saponin levels till reaching Titicaca cv. levels (Figure 4). The statistical analysis showed that both the year and the cultivar, played a significant role in determining saponin content (*p* < 0.05), but not the interaction between the two factors (*p* = 0.075) (Supplementary Table 3).

### Protein Content and Amino Acids

**FIGURE 5:**
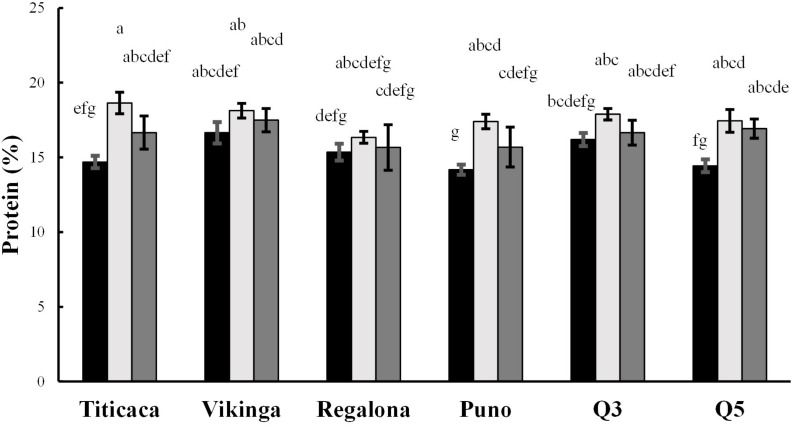
Protein contents. Protein content presented as percentage of protein per seed dry weight. Black bars represent 2017 values, light gray bars show 2018 values, and 2019 values are represented by dark gray bars. Bars that do not share the same letters show statistically significant differences, following the ANOVA test and *post-hoc* test Tukey.

Total protein content ranged from 13.8 to 19.1% of seed weight (Figure 5). The year, cultivar, and year x cultivar interaction factored in the determination of the protein content (p < 0.05) (Supplementary Table 3). On average, protein content was significantly higher in Vikinga cv. seeds compared to Q5 cv., Regalona cv., or Puno cv. Seeds harvested in 2018 showed higher protein content compared to the other two years of cultivation (Figure 5). Besides, differences were found within the same variety among growing seasons (e.g., Titicaca cv., Puno cv., and Q5 cv. harvested in 2017 showed lower content than the harvesting of 2018) and within the same year among varieties (e.g., in 2017, Vikinga cv. protein content was significantly higher than the content found in Puno cv.).

Regarding the amino acid profile, in all samples, glutamic acid was the most abundant amino acid (21.6 mg/g of seed, on average), followed by arginine and aspartic acid, while tryptophan showed the lowest concentrations (0.7 mg/g of seed) followed by cysteine and methionine (Supplementary Figure 2). The year of cultivation was a determining factor for all amino acid contents (*p* < 0.05), but the cultivar was not a significant factor in the case of threonine, proline, tyrosine, methionine, tryptophan, phenylalanine, and isoleucine, and the interaction between these factors only influenced the aspartic acid, cysteine, and arginine contents. Generally (except for methionine, cysteine, and tryptophan), 2018 yielded higher amino acid concentrations and 2019 the lowest. When comparing cultivars, Vikinga cv. and Titicaca cv. showed higher concentrations; on the contrary, lower amino acid concentrations were found in Puno cv., Q3 cv., and Q5 cv. However, cysteine and tryptophan concentrations were higher in 2017 seeds compared to 2018 seeds, and cysteine and methionine contents were higher in Puno cv. and Q3 cv. seeds compared to Vikinga cv. or Titicaca cv. (Table 1). In the case of proline, a higher content was found in Titicaca cv., Vikinga cv., Regalona cv., and Puno cv. seeds harvested in 2017 (6.2–8.2 mg/g of seed), and lower in 2019 and in Q3 cv. and Q5 cv. seeds harvested in 2017 (2.5–4.0 mg/g of seed) (Table 1).

**TABLE 1:** Amino acid profile.

Year	Cultivar	Aspartic acid	Glutamic acid	Serine	Histidine	Glycine	Threonine	Arginine	Alanine	Proline	Tyrosine	Valine	Methionine	Cysteine	Isoleucine	Tryptophan	Leucine	Phenyla lanine	Lysine
2017	Titicaca	11.84±0.93	21.37±2.1	6.5±0.73	4.44±0.31	7.93±0.57	5.25±0.44	11.83±1.1	6.34±0.48	7.29±1.31	4.22±0.47	6.15±0.46	1.71±0.89	1.78±0.16	5.65±0.49	0.84±0	9.16±0.68	5.8±0.44	7.85±0.59
		abcd	abcd	ab	abcd	abcd	abcdef	abcd	abcdef	abc	abc	abc	ab	abcd	abcd	ab	abcde	abcde	abc
	Vikinga	13.02±0.66	23.64±1.2	6.99±0.37	4.91±0.28	8.76±0.5	5.67±0.3	13.68±0.67	7.09±0.38	7.12±0.8	4.84±0.15	6.92±0.41	1.99±0.89	1.95±0.15	6.36±0.44	0.82±0	10.06±0.43	6.39±0.29	8.76±0.42
		abcd	abcd	ab	abcd	abcd	abcdef	abcd	abcdef	abc	ab	abc	ab	abcd	abcd	ab	abcde	abcde	abc
	Regalona	12.2±0.59	21.39±0.78	6.53±0.26	4.36±0.22	8.06±0.8	5.28±0.24	12.1±0.5	6.49±0.24	7.17±1.18	4.39±0.22	6.44±0.32	1.93±0.56	1.61±0.14	5.91±0.21	0.77±0	9.38±0.34	5.89±0.28	7.96±0.41
		abcd	abcd	ab	abcd	abcd	abcdef	abcd	abcdef	abc	abc	abc	ab	abcd	abc	ab	abcde	abcde	abc
	Puno	11.25±0.25	20.35±0.51	6.23±0.13	4.2±0.11	7.54±0.21	4.94±0.1	11.56±0.32	5.92±0.11	6.23±1.1	4.13±0.1	5.6±0.08	2.02±0.54	1.96±0.09	5.14±0.06	0.75±0	8.69±0.2	5.45±0.1	7.35±0.19
		d	abcd	b	abcd	cd	f	abcd	de	abc	bc	c	ab	ab	cd	ab	bde	bde	c
	Q3	12.23±0.21	22.29±0.5	7.12±0.19	4.29±0.14	8.12±0.07	5.5±0.11	11.93±0.26	6.66±0.15	4.01±1.45	4.23±0.17	5.93±0.08	1.43±0.33	2.14±0.04	5.4±0.07	0.79±0	9.34±0.2	5.82±0.09	8.12±0.12
		bcd	abcd	ab	cd	cd	ade	abcd	abc	abc	bc	bc	ab	a	abcd	ab	abcde	abcde	bc
	Q5	10.63±0.49	18.89±0.97	6.22±0.18	3.73±0.19	7.33±0.34	4.97±0.21	10.5±0.74	5.76±0.27	3.19±1.79	3.83±0.17	5.09±0.2	1.27±0.27	1.92±0.1	4.56±0.18	0.91±0	9.16±0.68	5.24±0.26	7.58±0.39
		d	ad	b	ab	ad	cdef	cd	aef	bc	c	c	ab	abc	d	a	abcde	cde	abc
2018	Titicaca	14.7±0.58	25.87±1.12	7.57±0.45	5.15±0.26	9.81±0.47	6.17±0.18	15.18±0.78	7.61±0.33	7.68±1.75	5.11±0.15	7.7±0.57	1.84±1.05	1.32±0.19	7.07±0.49	0.72±0	11.1±0.46	7.05±0.32	9.68±0.42
		abc	bc	ab	ab	bc	a	ab	bcd	ab	a	abc	ab	abcde	abcd	ab	ab	ab	ab
	Vikinga	14.37±0.49	25.8±1.95	7.87±0.48	5.23±0.25	9.71±0.57	6.06±0.1	15.31±1.82	7.34±0.48	6.92±1.48	5.15±0.65	7.22±0.49	1.56±0.13	1.71±0.11	6.91±0.35	0.78±0.06	10.74±0.82	6.72±0.49	9.65±0.5
		ab	abcd	ab	abcd	abcd	bc	abcd	abcdef	abc	abc	abc	a	abcd	ac	ab	abcde	abcde	abc
	Regalona	12.68±0.74	23.22±1.6	7.01±0.28	4.54±0.34	8.97±0.56	5.63±0.29	13.49±1.06	6.7±0.44	8.28±0.17	4.58±0.19	6.84±0.6	1.51±0.64	1.49±0.09	6.25±0.56	0.61±0	9.88±0.58	6.23±0.41	8.77±0.56
		abcd	abcd	ab	abcd	abcd	abcdef	abcd	abcdef	a	abc	abc	ab	bcde	abcd	ab	abcde	abcde	abc
	Puno	12±0.29	21.81±0.45	6.56±0.33	4.5±0.11	8.27±0.14	5.26±0.17	13.08±0.2	6.27±0.13	6.29±1.34	4.54±0.16	6.33±0.11	2.32±0.92	1.61±0.18	5.78±0.08	0.58±0	9.32±0.23	5.93±0.13	8.21±0.18
		bcd	abcd	ab	ac	cd	cdef	ac	cdef	abc	abc	ab	ab	abcde	ab	ab	abcde	abcde	abc
	Q3	11.99±0.18	20.73±0.3	6.98±0.19	4.03±0.05	8.14±0.14	5.47±0.1	11.72±0.18	6.49±0.09	6.22±1.65	4.57±0.12	6.27±0.07	2.42±0.45	2.06±0.19	5.7±0.07	0.59±0	9.46±0.19	5.96±0.1	8.19±0.12
		bcd	cd	ab	bd	cd	ade	bd	cf	abc	abc	ab	ab	abcd	ab	ab	ad	ad	bc
	Q5	13.57±0.2	22.87±0.14	7.58±0.05	4.59±0.1	9.03±0.11	5.86±0.05	13.11±0.33	6.94±0.08	7.17±2.1	4.68±0.19	6.43±0.09	1.47±0.74	1.45±0.27	5.82±0.1	0.55±0	10.03±0.11	6.36±0.12	8.98±0.12
		a	ab	a	abcd	ab	acd	abc	ab	abc	abc	a	ab	abcde	ab	b	ac	ac	a
2019	Titicaca	10.77±0.4	20.43±1.7	6.38±0.51	3.55±0.94	7.48±0.69	5.01±0.5	10.5±0.3	5.7±0.67	3.17±2.33	3.67±0.53	5.32±1.37	0.72±0.51	0.85±0.15	4.89±0.8	0.72±0.18	8.03±1.23	5.42±0.56	7.51±0.9
		d	abcd	ab	abcd	abcd	abcdef	d	aef	bc	abc	abc	ab	e	abcd	ab	abcde	abcde	abc
	Vikinga	12.07±1.96	19.59±4.69	6.96±1.02	4.16±1.11	7.91±1.31	4.41±1.17	10.86±2.32	6.16±0.9	3.13±2.55	3.41±0.81	5.74±1.04	1.71±1.14	1.52±0.43	3.91±1.83	0.67±0.06	8.94±1.79	5.05±1.01	7.82±1.56
		abcd	abcd	ab	abcd	abcd	abcdef	abcd	abcdef	bc	abc	abc	ab	abcde	abcd	ab	abcde	abcde	abc
	Regalona	11.09±1.44	19.57±4.03	6.47±0.95	3.51±1.14	7.36±0.86	4.62±1	11.11±1.69	5.72±0.85	2.53±1.94	3.9±0.46	5.43±0.91	0.99±0.92	1.34±0.12	4.6±0.9	0.68±0.3	7.86±1.95	5.3±0.87	6.79±1.76
		abcd	abcd	ab	abcd	abcd	abcdef	abcd	abcdef	c	abc	abc	ab	cde	abcd	ab	abcde	abcde	abc
	Puno	11.03±0.43	20.23±1.41	6.06±0.33	3.58±0.46	7.02±0.26	4.65±0.36	11.15±0.73	5.38±0.28	3.11±1.55	3.46±0.36	4.97±0.54	0.83±0.6	1.35±0.29	4.76±0.15	0.8±0.32	8.07±0.02	5.26±0.14	6.87±0.81
		d	abcd	ab	abcd	d	abcdef	cd	aef	bc	abc	abc	ab	abcde	d	ab	be	e	abc
	Q3	11.93±0.31	21.27±0.9	6.58±0.21	3.75±0.39	7.36±0.33	5.04±0.12	11.36±0.55	6.05±0.1	3.08±1.89	3.79±0.45	5.73±0.25	0.61±0.3	1.23±0.05	5.04±0.22	0.54±0.25	8.77±0.29	5.42±0.2	7.7±0.29
		cd	abcd	ab	abcd	ad	ef	cd	cdef	bc	abc	abc	ab	de	bd	ab	cde	cde	abc
	Q5	11.21±0.2	19.62±0.62	6.42±0.51	3.69±0.55	7.61±0.48	4.83±0.16	10.91±0.39	6.01±0.16	3.45±1.52	3.97±0.13	5.73±0.14	0.69±0.16	1.38±0.2	4.94±0.43	0.88±0.41	8.4±0.17	5.37±0.15	7.84±0.51
		d	ad	ab	abcd	abcd	ef	d	cdef	abc	c	bc	b	abcde	abcd	ab	e	de	abc

*Mean ± SD amino acid contents of samples are expressed as mg/g of seed weight. Statistical analysis following a One-way ANOVA test with a post-hoc Tukey test (proline), a Krustal-Wallis test by ranks (tryptophan), or a Welch’s ANOVA test with a Games-Howell post-hoc test, was performed. Different letters under each amino acid content show statistically significant differences between samples.*

### Mineral Content

The total content of phosphorous (P), potassium (K), calcium (Ca), magnesium (Mg), sodium (Na), copper (Cu), iron (Fe), manganese (Mn), and zinc (Zn) in quinoa seeds were determined in order to analyze the effect of genotype and environment on the mineral content (Table 2).

**TABLE 2:** Mineral seed contents.

Year	Cultivar	P (%)	K (%)	Ca (%)	Mg (%)	Na (ppm)	Fe (ppm)	Cu (ppm)	Mn (ppm)	Zn (ppm)
**2017**	**Titicaca**	0.36±0.02	1.44±0.12	0.19±0.04	0.21±0.01	39.33±12.50	76.30±7.34	6.24±1.45	34.63±2.20	26.33±4.08
		cdefgh	ab	abc	b	ab		Cu (ppm)	defgh	bcdef
	**Vikinga**	0.44±0.01	1.24±0.02	0.17±0.02	0.24±0.01	51.33±14.05	97.27±4.45	9.94±2.56	38.73±2.12	31.07±5.51
		ab	bh	abc	ab	ab		abc	def	abcdef
	**Regalona**	0.45±0.01	1.46±0.04	0.19±0.02	0.24±0.01	46.80±5.81	90.07±12.01	9.02±0.81	36.13±1.72	26.40±3.99
		ab	a	abc	ab	ab		abc	defgh	bcdef
	**Puno**	0.31±0.02	1.19±0.02	0.15±0.03	0.21±0.01	45.43±3.48	79.13±4.06	8.83±1.88	31.20±1.35	24.97±5.65
		gh	bi	bc	b	ab		abc	efghi	abcdef
	**Q3**	0.43±0.01	1.29±0.03	0.18±0.03	0.23±0.02	45.97±9.35	90.60±10.61	11.86±2.03	47.17±2.38	31.00±5.65
		ab	ab	abc	ab	ab		abc	bc	abcdef
	**Q5**	0.42±0.01	1.28±0.02	0.17±0.05	0.21±0.01	59.23±2.59	73.17±8.22	12.87±2.61	40.23±1.68	25.47±4.04
		abc	ab	abc	b	a		ab	cde	bcdef
**2018**	**Titicaca**	0.45±0.01	1.05±0.06	0.19±0.01	0.23±0.01	47.20±3.78	95.30±12.57	8.74±1.56	37.50±1.51	39.27±3.54
		ab	cdghijk	abc	ab	ab		abc	defg	abcde
	**Vikinga**	0.44±0.03	0.93±0.04	0.25±0.02	0.25±0.02	47.87±10.72	102.97±5.49	11.37±2.7	63.37±2.21	47.40±2.08
		abcd	dg	ab	ab	ab		abc	b	a
	**Regalona**	0.46±0.02	0.98±0.02	0.18±0.05	0.24±0.02	44.03±11.98	77.97±6.89	11.46±3.24	36.43±1.81	40.70±3.63
		a	cdefg	abc	ab	ab		abc	defg	abcd
	**Puno**	0.39±0.02	1.00±0.03	0.23±0.02	0.24±0.02	48.23±7.02	91.37±17.25	12.53±1.59	30.50±2.49	37.67±2.46
		bcdef	cdfg	abc	ab	ab		abc	fghi	abc
	**Q3**	0.43±0.03	1.00±0.06	0.22±0.06	0.25±0.02	47.00±13.86	91.57±7.50	14.27±3.71	72.07±0.99	41.13±1.37
		abcde	dfgjk	abc	ab	ab		a	a	ab
	**Q5**	0.34±0.02	0.96±0.08	0.16±0.03	0.22±0.02	46.73±12.71	95.67±6.92	9.10±1.87	46.67±1.72	35.57±2.97
		efgh	fgik	abc	b	ab		abc	bcd	abcde
**2019**	**Titicaca**	0.35±0.03	1.43±0.12	0.26±0.05	0.23±0.01	58.53±2.81	100.37±16.98	7.21±1.20	35.17±5.33	20.50±2.09
		abcdefgh	abe	ab	ab	a		bc	defgh	f
	**Vikinga**	0.32±0.01	1.27±0.03	0.27±0.06	0.27±0.02	49.85±6.15	124.40±39.17	8.37±0.33	35.60±4.38	21.30±0.28
		fgh	abe	a	a	ab		abc	defg	def
	**Regalona**	0.36±0.03	1.40±0.08	0.16±0.04	0.22±0.01	39.50±1.57	77.93±8.36	8.91±0.99	22.57±2.67	22.17±0.55
		abcdefgh	abc	abc	b	b		abc	i	def
	**Puno**	0.33±0.01	1.30±0.08	0.24±0.04	0.23±0.02	60.60±17.58	82.27±6.69	8.35±1.54	29.20±4.62	22.23±2.77
		efgh	abcd	abc	ab	ab		abc	ghi	ef
	**Q3**	0.34±0.01	1.17±0.04	0.13±0.01	0.22±0.01	35.50±1.73	83.67±5.76	8.95±0.66	29.10±0.75	26.43±1.63
		defgh	bij	c	b	b		abc	hi	cdef
	**Q5**	0.34±0.02	1.26±0.12	0.21±0.06	0.22±0.01	63.87±16.94	94.30±4.20	12.58±1.88	39.47±1.86	21.90±5.02
		bcdefg	abe	abc	ab	ab		ab	def	abcdef

*Mean±SD mineral contents are presented as percentage of seed weight (P, K, Ca, and Mg) or as parts per million (Na, Fe, Cu, Mn, and Zn). Statistical analysis following a One-way ANOVA test with a post-hoc Tukey test (Ca, Mg, and Cu contents), a Krustal-Wallis test by ranks (Mn content), or a Welch’s ANOVA with a Games-Howell post-hoc test (P, K, Na, Fe, and Zn contents), was performed. Different letters under each mineral content show statistically significant differences between samples.*

Overall, it was observed that mineral content was greatly influenced by the year of cultivation and by the cultivar (*p* < 0.05), except for Na (p = 0.419 and p = 0.063, respectively), and by the interaction between the two factors (except for Mg, Fe, and Zn content) (Supplementary Table 3). In the case of P, Cu, Mn, and Zn (Table 2), contents were higher in seeds harvested in 2018, with a difference especially remarkable in Zn. On the other hand, this trend was inverted in the case of K, and the contents of Ca, Mg, and Fe were significantly lower in 2017 seeds (Table 2). For P, Mg, Fe, Mn, and Zn contents, Puno cv. seeds showed the lowest levels while Vikinga cv. showed the highest contents, while for K, the content was higher in Titicaca cv. and Regalona cv.

### Antioxidant Capacity

**FIGURE 6:**
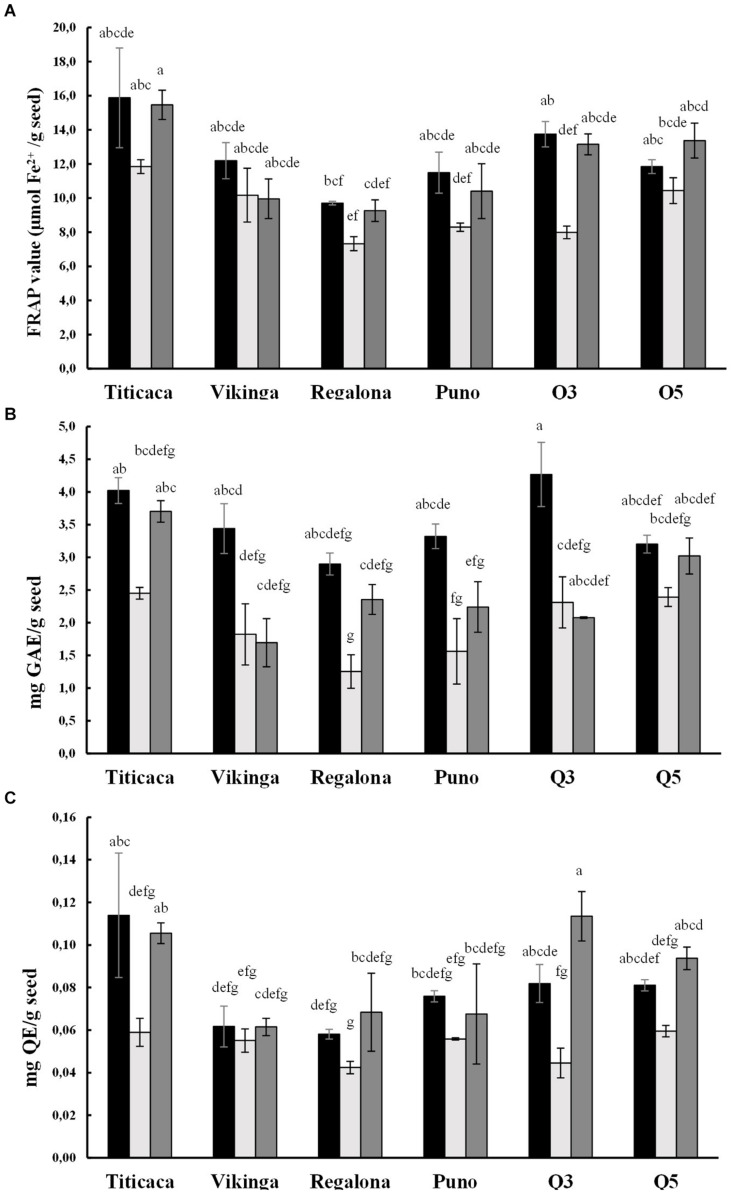
Antioxidant capacity of quinoa seeds. Black bars represent 2017 values, light gray bars show 2018 values, and 2019 values are represented by dark gray bars. **(A)** Antioxidant power of quinoa seeds was measured using the ferric reducing antioxidant power (*FRAP*) assay and is expressed as μmol of Fe^2+^per gram of seed. Statistical differences were analyzed through a Welch’s ANOVA test followed by a Games-Howell post-hoc test. **(B)** Total polyphenol content (TPC) is expressed as milligrams of gallic acid equivalents (GAE) per gram of seeds. The statistical analysis performed was a One-way ANOVA test followed by a post-hoc Tukey test. **(C)** Total flavonoid content (TFC) is expressed as milligrams of quercetin equivalents (QE) per gram of seeds. A Krustal-Wallis test by ranks was performed for multiple comparisons. Bars that do not share the same letters show statistically significant differences.

We evaluated the antioxidant capacity by performing the FRAP assay, together with the quantification of total polyphenols (TPC) and flavonoids (TFC) contents (Figure 6). These three variables showed strong correlations among them (p = 0,000, Supplementary Figure 4) and a significant influence of the year of cultivation, the cultivar (p < 0.05), and their interaction in the case of FRAP value and TFC.

Significant differences were found when comparing cultivars. Titicaca cv. showed the highest antioxidant capacity, TPC, and TFC, followed by Q5 cv., while Regalona cv. presented the lowest values (Figure 6). The lowest FRAP, TPC, and TFC values appeared in the seed samples harvested in the second year (2018), while samples from the 2017 season showed the highest. When analyzing changes within the same variety, it was observed that Q3 cv. seeds harvested in 2017 presented the highest phenolic content and those harvested in 2019 had the largest flavonoid content, although in this last year, Q3 cv. seeds showed an especially low phenolic content.

### Color

The color was determined as parameters related to the technological and functional quality of the seeds (Guiotto et al., 2020). In this analysis, the color values indicated that there were significant differences when comparing cultivars or among year of sowing. Both factors and their interaction had a significant influence on luminosity (L^∗^) and in hue value (h°), but only the cultivar factored in chroma (C^∗^, color saturation) and color distance (ΔE). In general, Regalona cv. and Puno cv. were the clearest (high luminosity (L^∗^), Table 3). This characteristic was significantly affected by the year of cultivation, being 2018 the year associated with darker seeds. Regarding cultivars, the a^∗^ parameter, that corresponds to red (+a^∗^) or green (-a^∗^) component of color, Titicaca cv. and Q5 cv. seeds were the reddest (Table 3 and Supplementary Figure 3). Although there were slight differences, 2018 was the year that yielded higher a^∗^ values. The b^∗^ component of the color (+b^∗^ yellow, -b^∗^ blue) ranged between 18.23 and 27.43, being Q5 cv., followed by Titicaca cv., Regalona cv., and Puno cv., the cultivars that yielded seeds associated with a highest yellow component.

**TABLE 3:** Seed color-related parameters.

Year	Cultivar	L*	a*	b*	h°	C*	Δ E
**2017**	**Titicaca**	56.3±1.5	5.5±0.4	24.2±0.5	77.3±1	24.8±0.5	1.2±1.4
		abcde	defgh	ac	abcd	b	abc
	**Vikinga**	59.8±1.1	4.9±0.2	20.6±0.8	76.7±0.6	21.2±0.8	6.1±0.4
		abc	gh	abcd	abc	cdef	bc
	**Regalona**	61.4±1.7	4.3±0.3	21.5±1	78.7±0.8	21.9±1	7±1.4
		abc	hi	abcd	a	bcdef	abc
	**Puno**	61.2±3.7	3.6±0.5	20.4±1.1	80.1±1.7	20.7±1.1	7.9±2.3
		abcde	i	abcd	abcd	def	abc
	**Q3**	55.6±2.1	5.2±0.7	20.4±0.3	75.6±2	21.1±0.3	4.7±0.5
		abcde	fgh	bd	abcd	cdef	bc
	**Q5**	56±0.7	6.2±0.7	27.4±1.5	77.2±0.6	28.1±1.6	3.1±1.4
		cd	abcdef	ab	abc	a	abc
**2018**	**Titicaca**	52.3±1	6.8±0.4	20.8±1.2	71.7±0.2	21.9±1.3	5.3±1.3
		de	bc	abcd	d	bcdef	abc
	**Vikinga**	59.6±1	5.9±0	22.5±1.3	75.2±0.8	23.3±1.2	5±0.2
		abc	adefg	abc	abcd	bcde	bc
	**Regalona**	62.1±0.6	5.3±0.3	23.2±0.4	77.3±0.4	23.8±0.4	6.9±0.7
		ab	fgh	ac	ab	bc	abc
	**Puno**	61.4±2.1	5.3±0.5	23.1±0.5	77±1	23.7±0.5	6.3±2
		abcde	efgh	ac	abcd	bcd	abc
	**Q3**	50.7±0.9	5.2±0.1	18.4±0.5	74.1±0.6	19.1±0.5	7.9±1
		e	fgh	d	cd	f	ab
	**Q5**	52.5±1.9	7±0.2	22.9±1	73.1±1.2	24±0.9	3.9±2
		bcde	ab	abcd	bcd	bc	abc
**2019**	**Titicaca**	50.3±3.7	6.7±0.6	21.7±2	72.6±2.7	22.7±1.7	6.1±4.1
		abcde	abcd	abcd	abcd	bcde	abc
	**Vikinga**	55.4±1.7	5.3±0.2	19.6±1.3	74.9±0.6	20.3±1.3	5.3±1.1
		abcde	bdefgh	cd	bcd	ef	abc
	**Regalona**	64.6±0.4	4.7±0.4	24.1±0.9	79.1±0.6	24.5±1	9.3±0.5
		a	ghi	abc	a	b	a
	**Puno**	63.1±0.9	4.3±0.5	22.5±0.9	79.1±0.8	23±1	8.1±1.2
		a	hi	abcd	a	bcde	abc
	**Q3**	56.8±2.7	5.4±0.5	22.1±0.5	76.4±0.8	22.7±0.6	3.7±0.9
		abcde	efgh	abc	abcd	bcde	bc
	**Q5**	54.1±1.7	6.6±0.4	23.9±0.5	74.7±1.1	24.8±0.4	2.2±1.2
		bcde	abcde	ac	abcd	b	c

Mean±SD is given for each color parameter of the CIELAB and CIELCh color spaces. L*: Lightness from black (0) to white (100). a*: from green (-) to red (+) component. b*: blue (-) to yellow (+) component. h°: hue angle. C*: chroma (color saturation). ΔE: color difference. ΔE values are calculated relative to Titicaca cv. 2017 seeds. Different letters underneath indicate statistically significant differences performing a One-way ANOVA followed by a Tukey post-hoc test (a* and C*) or a Welch‘s ANOVA test followed by a Games-Howell post-hoc test (L*, b*, h°, and ΔE).

### Correlation, Linear Regression, Path Analysis, and Principal Components Analysis (PCA)

A Pearson’s correlation coefficient test was performed to analyze the correlation between variables (Supplementary Figure 4). All amino acids contents (except for cysteine and tryptophan contents) showed high correlation coefficients with total protein content and among them, besides showing high positive correlation with P and Zn contents and negative correlations with flavonoid and K contents, yield, seed viability, and germination rates. Flavonoid content also correlated with the phenolic content and the antioxidant capacity (r = 0.87), and with yield, germination, and seed viability rate, as well as with color parameters and some mineral contents, especially Zn (r = −0.6). Both, germination rate and seed viability presented high correlation coefficients with each other (r = 0.81) and with yield (r = 0.69). Seed viability and germination rate correlated negatively with protein (r = −0.65 and r = −0.58, respectively) and amino acids contents and with all the minerals except for Na. The correlation coefficients of Zn and K contents with seed viability (r = −0.71 and r = 0.75, respectively) and germination rate (r = −0.59 and r = 0.62, respectively) were also remarkable.

This analysis was followed up by simple linear regressions to evaluate the influence of yield on different seed nutritional quality-related traits (Table 4). Seed viability showed the largest linear correlation (R^2^ = 0.455) with yield, where yield explains 67.5% of the viability rate’s variance. K content also showed a large relation with yield (R^2^ = 0.293), being this trait responsible for 54.2% of the K content variance. The model obtained relating yield to total protein content (R^2^ = 0.289) predicted a decrease in seed protein content of 0.12% when the cultivar yield increases in 1 t/ha. Other qualitative seed traits such as germination rate, antioxidant capacity and flavonoid, phenols, Mg, Fe, and Zn contents proved to be influenced by yield, producing adequate linear models (p < 0.05). However, seed weight and P, Ca, Na, and Cu contents could not be explained by yield performance (p > 0.05).

**TABLE 4:** Regression analysis summary of predictive models for yield predicting qualitative seed variables.

	Unstandardized coefficients		Standardized coefficients		R^2^	F	p
Model	b	SE	β	p			
SW = b_1_ + Yield*b_2_					0.000	0.004	0.952
VR = b_1_ + Yield*b_2_					0.455	43.440	0.000
Intercept	−1.193	7.908		0.881			
Yield	23.689	3.594	0.675	0.000			
GR = b_1_ + Yield*b_2_					0.133	7.952	0.007
Intercept	0.475	0.071		0.000			
Yield	0.091	0.032	0.364	0.007			
TFC = b_1_ + Yield*b_2_					0.160	9.940	0.003
Intercept	0.054	0.006		0.000			
Yield	0.009	0.003	0.401	0.003			
TPC = b_1_ + Yield*b_2_					0.213	14.043	0.000
Intercept	1.877	0.253		0.000			
Yield	0.430	0.115	0.461	0.000			
FRAP = b_1_ + Yield*b_2_					0.152	9.308	0.004
Intercept	9.304	0.704		0.000			
Yield	0.976	0.320	0.390	0.004			
Protein = b_1_ + Yield*b_2_					0.289	21.174	0.000
Intercept	2.868	0.057		0.000			
Yield	−0.119	0.026	−0.538	0.000			
P = b_1_ + Yield*b_2_					0.010	0.540	0.466
K = b_1_ + Yield*b_2_					0.293	21.593	0.000
Intercept	1.010	0.046		0.000			
Yield	0.098	0.021	0.542	0.000			
Ca = b_1_ + Yield*b_2_					0.023	1.175	0.283
Mg = b_1_ + Yield*b_2_					0.151	9.224	0.004
Intercept	0.243	0.005		0.000			
Yield	−0.007	0.002	−0.388	0.004			
Na = b_1_ + Yield*b_2_					0.015	0.755	0.389
Fe = b_1_ + Yield*b_2_					0.122	0.725	0.010
Intercept	99.342	4.080		0.000			
Yield	−4.979	1.854	−0.349	0.010			
Cu = b_1_ + Yield*b_2_					0.000	0.019	0.892
Mn = b_1_ + Yield*b_2_					0.064	3.488	0.068
Intercept	45.421	3.670		0.000			
Yield	−3.087	1.653	−0.253	0.068			
Zn = b_1_ + Yield*b_2_					0.208	13.670	0.001
Intercept	37.765	2.285		0.000			
Yield	−3.839	1.038	−0.456	0.001			

*Simple linear regression analyses were performed. SE, standard error; SW, seed weight; VR, Viability rate; GR, germination rate; TFC, total flavonoid content; TPC, total phenolic content; FRAP, antioxidant capacity.*

Going further, path analysis was performed to define the direct and indirect contributions of each trait on seed germination rate. First, a predictive multiple linear regression model was performed following the stepwise method in order to find seed traits with a direct effect on germination rates. Individual amino acids contents were not included in the analysis because of their high multicollinearity. Yield, antioxidant capacity, phenols, flavonoids, protein, Zn, Fe, Ca, and Zn contents were eliminated from the model, while Mg, K, and Mn contents, entered the model as first-order predictors, explaining 55.8% of the germination rate variability (Table 5) and generating the following model:

$$\begin{array}{c} \left[\mathrm{Germination}\mathrm{rate}\right]=1.137+0.585\left[\text{K}\right]-4.141\left[\text{Mg}\right]\\ -0.006\left[\text{Mn}\right] \end{array}$$

**TABLE 5:** Direct effects of predictor variables of first-, second-, third-, and fourth-order on germination rate, tolerance and variance inflation factor of the path analysis.

Response variable	Predictor varibles	Adjusted R^2^	Direct effect	Tolerance	VIF
GR	K	0.558	0.430	0.771	1.297
	Mg		−0.300	0.852	1.174
	Mn		−0.282	0.681	1.468
K	Zn	0.646	−0.682	0.865	1.156
	TPC		0.256	0.865	1.156
Mg	Ca	0.498	0.615	1.000	1.000
	TFC		−0.382	1.000	1.000
Mn	Zn	0.507	0.609	0.742	1.348
	Ca		0.270	0.965	1.036
	TPC		0.293	0.837	1.195
	Cu		0.242	0.817	1.224
Zn	TFC	0.434	−0.457	0.841	1.190
	Protein		0.348	0.841	1.190
TPC	TFC	0.406	0.489	0.836	1.197
	Yield		0.282	0.836	1.197
Ca	Fe	0.136	0.391	1.000	1.000
Cu	FRAP	0.131	−0.385	1.000	1.000
Protein	Yield	0.353	−0.432	0.871	1.148
	Fe		0.309	0.871	1.148

*Subsequent multiple linear regression analyses were performed. GR, germination rate; TFC, total flavonoid content; TPC, total phenolic content; FRAP, antioxidant capacity.*

(being the germination rate expressed as %, K and Mg content as % of seed weight, and Mn content as ppm of dry seed). K content showed a strong positive effect in the germination rate, accounting for 43% of the germination rate, while Mg, and Mn contents were in turn responsible for 30 and 28.2% of the germination rate’s variance, respectively, presenting a negative effect. Going further, TFC and Ca contents acted as second-order predictors of the germination rate through Mg content, having the earlier a negative effect on Mg and the latter a strong positive effect (61.5% of Mg’s variance). Ca was also a second-order predictor through Mn content, together with Zn, Cu, and phenols contents. Zn showed the largest direct effect on Mn (60.1% of Mn’s variance, Table 5). Through the K content path, two second-order predictors were found, phenols and Zn contents, showing the latter a strong negative contribution to K’s variance (68.2%, Table 5). The analysis also showed third-order predictors like Fe content (through Ca content), TFC (through a positive effect on TPC and a negative one on Zn), yield (through TPC), total protein content (through Zn), and antioxidant activity (via negative influence on Cu), as well as two fourth-order predictors, Fe content and Yield, which explained protein content with a positive effect of 30.9% and a negative effect of 43.2%, respectively, on protein’s variance (Table 5). No collinearity was found in the analysis.

**FIGURE 7:**
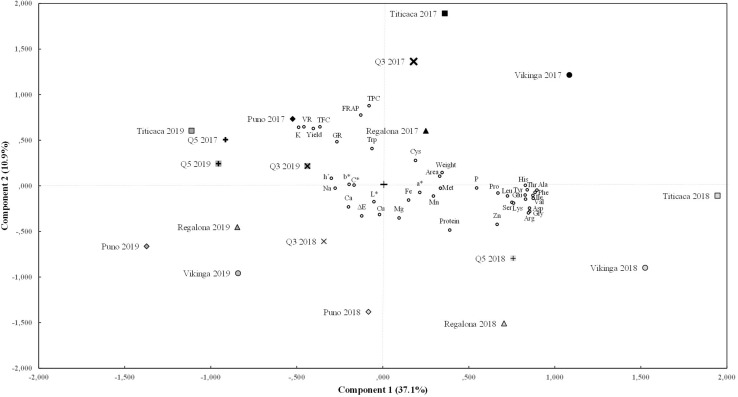
Principal components analysis. Biplot of main components 1 and 2 for the cultivars sown in each year of the experiment and for the variables tested. Component 1 (X axis) is contributed mainly by seed viability rate (VR) and yield, amino acid content (except methionine, cysteine, and tryptophan), P, K, and Zn contents. Component 2 (Y axis) includes yield, viability and germination rate (GR), FRAP value, and protein, tryptophan, polyphenols (TPC), flavonoids (TFC), K, and Zn contents.

Furthermore, a principal component analysis (PCA) was performed to reduce the number of variables (Figure 7). This analysis identified five principal components that were able to explain 69% of the variance. Component 1, which contributed to 37% of the variance, was mainly explained by the amino acid contents (except for cysteine, methionine, and tryptophan), yield, seed viability, and by the content of some minerals such as P, K, and Zn. In line with this, a strong positive correlation was found between the content of amino acids, P and Zn contents, which have high component 1 values, and a negative correlation of these variables with yield, seed viability, or K, with low component 1 values (Supplementary Figure 4 and Figure 7). Component 2 (explaining 11% of the variance) was accounted mainly for the antioxidant parameters (FRAP value, TPC, and TFC), yield, seed viability, the germination rate, and K content (high component 2 values), and for protein and Zn content, which correlated negatively with the other variables and showed low component 2 values (Supplementary Figure 4 and Figure 7). Component 3 (explaining 9% of the variance) considered the color parameters L^∗^, a^∗^, h°, the FRAP value, and the protein content. The color parameters C^∗^ and b^∗^ together with the Ca, Mg, and Fe contents and the germination rate, contributed to component 4 (explaining 6% of the variance). Component 5 (explaining 5% of the variance) included the content of P, Cu, Mn, and Zn, cysteine, and seed weight.

By reducing the number of variables to main components it was possible to classify the quinoa genotypes for each year of harvesting in different groups (Figure 7). As consistently found in previous analyses, the year of cultivation was the most important factor when grouping the different variables studied. Seeds that belong to the 2017 harvest (except Puno cv. and Q5 cv.) formed the first group, presenting positive values for component 1 (meaning higher amino acids, P, and Zn contents, and lower K content, yield, and viability) and positive values for component 2 (related to higher antioxidants contents, viability, germination rate, tryptophan, and K content, and lower protein and Zn content). All 2018 seeds belonged to the second group and showed negative values for component 2, which explains the higher protein and Zn content and lower antioxidant capacity, germination, viability, and yield found that year. This group also shows positive values (except Q3 cv. and Puno cv.) for component 1, explaining the higher amino acids contents. Meanwhile, all 2019 seeds, comprising the third group, presented negative values for component 1, which coincides with the lower amino acids’ concentrations found that year. However, 2017 seeds from Puno cv. and Q5 cv. appeared closer in the PCA analysis to the seeds from the 2019 harvest than other 2017 seeds, showing also lower amino acid contents. For component 1, 2018 samples showed higher values than 2019, and Vikinga cv. seeds showed higher levels than Regalona cv., Puno cv., Q3 cv., or Q5 cv. For component 2, 2018 samples were significantly lower than 2019, and 2017, and Vikinga cv., Puno cv., and Q3 cv. had lower values than Titicaca cv., Regalona cv., or Q5 cv. Vikinga cv. seeds from 2019 showed the lowest component 2 level for 2019 samples and one of the lowest overall, which coincides with its poor yield performance, and its low viability and germination rate, and flavonoid and polyphenol contents.

Differences among cultivars were also observed and were reflected in Figure 7 through the PCA. Titicaca cv. seeds presented the highest values for component 2, and, in 2018, for component 1, although with larger variations. These seeds showed higher yields, germination rates, and antioxidant contents, although they were negatively affected by the environment in 2018. In contrast, Vikinga cv. seeds showed higher values for component 1 in 2017 and 2018 but very low values for component 2 in 2018 and 2019, being the only cultivar with lower values in 2019 seeds than in 2018 seeds. This can be seen in their consistently amino acids contents and the steeped decrease in yield, seed germination rates and viability, and antioxidants contents in both 2018 and 2019. On the other hand, Puno cv. seeds values are low for both components and show low germination rates, seed weights, and protein, amino acids, and antioxidants contents.

## Discussion

In the last decade, quinoa has acquired an increased agronomical and nutritional relevance related to the capacity of adaptation of this crop to different environments together with the exceptional nutritional properties of their seeds, which include high protein contents, an optimal amino acid balance, and an excellent antioxidant capacity, this later largely related to the high phenol content (Bazile et al., 2016; Jacobsen, 2017; Angeli et al., 2020). However, the establishment of this crop in many agronomical areas outside South America is still limited. It could be considered that quinoa cultivar selection process remains unfinished for new cultivation areas, including those located in Southern Europe. Furthermore, although the potential of this crop has been comprehensively analyzed in nearby areas (Jacobsen, 2017) there is still very limited information regarding the stability of seed nutritional characteristics under changing environments.

Multiple environmental factors, such as temperature, water status, photoperiod and light quality, and soil nutrient content, together with genetic features, are responsible for determining the quality of seeds. Other factors like the physiological status of the plant during growth or postharvest parameters such as moisture and temperature during the storage of seeds may play pivotal roles in determining quality as well. Ultimately, genotype, environment, and their interaction are the main factors determining the status of seeds (Hakeem, 2015). In this study, six different cultivars were used in order to examine the effect of the genotype on different physiological and nutritional traits of quinoa seeds, which were sown in three consecutive years aiming to analyze the environmental effect on those parameters.

Saponins are antinutrients that can diminish the nutritional value of quinoa seeds. These compounds can alter the absorption of minerals such as Fe and Zn (Ruales and Nair, 1993) and they give bitterness to the seeds decreasing their palatability. Therefore, extensive efforts have been made through breeding programs toward reducing their concentrations in seeds (Mastebroek et al., 2000; Zurita-Silva et al., 2014). Nevertheless, the increasing evidence regarding their multiple bioactivities for health may be worth considering, such as anti-inflammatory, antitumor, hypocholesterolemic, or immunomodulatory (Marrelli et al., 2016; Singh et al., 2017; Navarro del Hierro et al., 2018; El Hazzam et al., 2020). The limit established to classify quinoa varieties as sweet or bitter is 0.11% of saponin per seed fresh weight (Koziol, 1991). Thus, Titicaca cv. (used in this study) can be classified as bitter, as had been previously reported (Medina-Meza et al., 2016), and, on the other hand, Vikinga cv. could be considered a “low saponin” variety (Figure 4; Medina-Meza et al., 2016). The results showed that the main difference in saponin contents was determined by the cultivar, although higher contents in 2019 Vikinga cv. seeds could be observed compared to previous years (Figure 4). As previously described, 2019 was the year that showed a reduced water supply (Supplementary Table 1 and Figure 1). This finding is in agreement with that of Reguera et al. (2018), who suggested that saponin content is mainly a genotypic-dependent trait, although it can change as well under stress conditions including drought or soil salinity (Gómez-Caravaca et al., 2012; Pulvento et al., 2012). In fact, according to Figure 4, it seems that specific environmental conditions might even lead to the production of bitter quinoa seeds but from sweet quinoa varieties. Furthermore, it should not be ruled out an influence of the genetic purity of the seeds used in this study in the variations observed in the saponin content, as it may reflect genetic segregation or genetic cross-contamination, which might as well explain the reduced Vikinga cv. seed yield observed in 2019. Therefore, further research to understand the mechanisms or environmental conditions that impact the content of saponins of quinoa seeds would be of interest. This would benefit future breeding efforts if they were to target, either to reach low-saponin quinoa seeds, or even saponin-enriched seeds as a selection trait, considering the current popularity of these phytochemicals as bioactive compounds.

Quinoa seeds are well known for possessing high protein contents. This study shows a protein range that went from 14.1 to 18.6% of seed weight, with an average of 16.5% protein per seed weight (Figure 5). These results were similar to those obtained in other works on quinoa (Koziol, 1992; Miranda et al., 2012; Reguera et al., 2018) and reflect higher protein contents compared to important staple cereal crops such as barley (11%), wheat (10%), maize (14%), or rice (8%) (Koziol, 1992; Nowak et al., 2016; Filho et al., 2017). However, not only protein quantity but also protein quality may impact human diet. Most proteins of plant origin have very low levels of essential amino acids, especially tryptophan, methionine, and lysine compared to those of animal origin (Friedman, 1996). Particularly, lysine and tryptophan are present in low levels in grains of cereal crops like barley and wheat, while sulfur amino acids (methionine and cysteine) show lower levels in legume seeds including soybean or beans (Friedman, 1996). In line with this, quinoa seeds are considered a “complete protein” source since they provide all amino acids essential for human consumption. They have higher levels of lysine, methionine, and cysteine compared to cereals or legumes which makes quinoa a great food complement in healthy diets (Koziol, 1992; Repo-Carrasco et al., 2003; Abugoch James, 2009; Filho et al., 2017). However, different studies have reported limiting essential amino acid contents in quinoa according to the daily requirements established by the Food Agriculture Organization (FAO) (World Health Organization and United Nations University, 2007; Gonzalez et al., 2012; Miranda et al., 2012; Präger et al., 2018; Craine and Murphy, 2020). In this work, all samples analyzed met the daily lysine and leucine requirements established by the FAO for all age groups (World Health Organization and United Nations University, 2007; Table 1). All samples from 2017 harvest and 2019 Puno cv. and Q5 cv. met the daily requirements of tryptophan for adults, but only 2017 Titicaca cv. and Q5 cv. seeds contained enough tryptophan to meet the requirements for children (World Health Organization and United Nations University, 2007; Table 1). The other samples did not meet the requirements of either group. In the case of sulfur amino acids (combining cysteine and methionine contents), only 2017 seeds of all cultivars and those from Puno cv. and Q3 cv. harvested in 2018 met the daily children and adults’ recommendations, especially due to the low levels of methionine (Table 1). Both methionine and tryptophan may be limiting in quinoa (Mahoney et al., 1975; Gonzalez et al., 2012; Craine and Murphy, 2020). Therefore, overall, and based on these and previous analysis (Craine and Murphy, 2020), increased lysine and sulfur amino acids levels should be breeding targets in quinoa toward enhancing quality to reach Food Security.

It has been hypothesized that protein and amino acids contents can be determined by nitrogen availability in the soil, the environmental and agroecological conditions, and the genotype (Thanapornpoonpong et al., 2008; Craine and Murphy, 2020). In this study, a significant variability throughout samples was found in both protein and amino acid contents (Figure 5 and Table 1). While the evidences presented by Miranda et al. (2012) suggest a strong influence of the genotype in the amino acids contents, the studies performed by Reguera et al. (2018) and Prieto et al. (2021) support the hypothesis that environmental factors can influence the protein content of quinoa seeds. Reguera et al. (2018) found differences in protein quantity among seeds harvested in different countries but not among cultivars in a certain location, while Prieto et al. (2021) showed an important increase in quinoa protein content when the crop underwent heat stress. In the present study, there is a strong influence in both protein and amino acids contents by the year of cultivation, being 2018 the growing season with higher contents except for cysteine and tryptophan (Figure 5 and Table 1). Some amino acids did not show significant changes among cultivars, but in most cases Vikinga cv. seeds showed higher contents and Puno cv. seeds lower. It is worth mentioning that Titicaca cv. seeds showed larger differences for most amino acids, being generally 2018 seeds richer, while Regalona cv. and Q3 cv. seeds had a more stable amino acid content among years of harvest. This differential response from different genotypes to changing environmental conditions had already been observed by Präger et al. (2018), who described higher essential amino acid contents in Jessie cv. and Zeno cv. in 2016 compared to 2015, but not in Puno cv. or Titicaca cv. Thus, the modulation of protein and amino acid contents by environmental conditions is largely dependent on the genotype. Understanding the mechanisms responsible for these changes in the amino acidic profile will be of importance in the study of stress tolerance in different quinoa cultivars since some essential amino acids also play a role as osmolytes. For instance, the accumulation of branched-chain amino acids (BCAAs) (valine, leucine, isoleucine) is induced by osmotic stress (Joshi et al., 2010).

Mineral content is an important determinant of seed quality. Quinoa seeds are characterized by presenting a high content of Ca, Mg, Fe, Cu, and Zn; moreover, Ca, Mg, and K are found in sufficient quantities in quinoa seeds to meet a balanced human diet, since they are in bioavailable forms (Repo-Carrasco et al., 2003). The seeds here analyzed met Fe daily requirements for all age groups, even for women at a menstruating age (World Health Organization, 2004), but K contents did not meet the requirements for pregnant or lactating women (Turck et al., 2016). The mineral contents here presented are similar to previous reports in quinoa (Table 2; Aranda et al., 2013; Prado et al., 2014; Reguera et al., 2018). In fact, Regalona cv. and Titicaca cv. levels matched in range with those reported by Reguera et al. (2018), who used these same varieties, but not for Na and Ca contents (higher in the present study). In the preceding study, stark differences were found between sowing locations, which were attributed to differences in soil composition (Reguera et al., 2018). In this study, both genotype and environment influenced mineral contents (except for Na), even though all seeds were sown at the same location in the three consecutive years. For instance, contents of P, Mg, Fe, Mn, and Zn were significantly higher in Vikinga cv. seeds and in 2018 seeds (Table 2). It should be noted that further analysis should be made to determine if the increased mineral content is correlated with mineral bioavailability and to determine the role of the genotype and environment regulating this aspect, considering the differences previously observed in quinoa (Vidueiros et al., 2015) and the effect of components like saponins or phytic acid on the mineral’s bioavailability in quinoa (Ruales and Nair, 1993).

Also, antioxidants may condition the nutritional quality and shelf life of seeds. From a nutritional point of view, these compounds add health benefits as they can reduce the risk of cancer and cardiovascular diseases (Vega-Gálvez et al., 2010; Tang and Tsao, 2017). In line with this, quinoa seeds are an excellent source of antioxidants, especially because of their high contents of phytochemicals like polyphenols, flavonoids, and vitamin E (Tang and Tsao, 2017), which also exceed the levels found in cereals (Gorinstein et al., 2007). The contents of these phytochemicals are variable in quinoa and are genotype-dependent. Besides, there is evidence of changes related to environmental constraints such as salt or water stress reflecting the environmental control in the synthesis of these compounds (Fischer et al., 2013; Aloisi et al., 2016; Ismail et al., 2016). In the present study, the amounts of polyphenols, flavonoids, and FRAP capacity were found at comparable levels to those reported by Ismail et al. (2016), Paśko et al., 2009, Abderrahim et al. (2015), and Fischer et al. (2013). Both genotype and environmental conditions were determinant factors of these parameters, being lower in seeds harvested in 2018 and in Regalona cv. and Puno cv. seeds, while higher in Titicaca cv. (Figure 6). Nonetheless, it should be highlighted that the response to different environmental conditions seems to differ between cultivars, and Titicaca cv. seeds have a steeper decrease in FRAP value and TFC compared to Vikinga cv. seeds (Figure 6). This supports the hypothesis that the antioxidant capacity depends on the genotype, the environment, and the interaction of these factors. Both, Ismail et al. (2016) and Aloisi et al. (2016) propose that those varieties more tolerant to stress require a lower production of antioxidants. For this reason, more research needs to be performed investigating the mechanisms responsible for the changes in the antioxidants of quinoa seeds. This would benefit future breeding efforts if they were to target antioxidant capacity as a selection trait.

The content of phenols and other secondary metabolites can affect seed parameters such as seed color (Ballester-Sánchez et al., 2019). Similarly, seed color might be indicative of the content of these compounds. In this study color differences were related to the cultivar, being Titicaca cv. and Q5 cv. seeds darker and redder, and Regalona cv. and Puno cv. seeds lighter (Table 3 and Supplementary Figure 3). The year of cultivation only affected the a^∗^ component of color (redness), being 2018 seeds the reddest. The L^∗^ component of color negatively correlated with FRAP and TPC levels (Supplementary Figure 4), i.e., darker seeds provided more antioxidants. This characteristic had been previously reported in quinoa seeds and in other plant species (Szydłowska-Czerniak et al., 2011; Abderrahim et al., 2015).

Yield is a common selection criterion for quinoa breeding programs, aiming to increase productivity (Zurita-Silva et al., 2014). The varieties cultivated in this study yielded between 0.7 and 3.25 t/ha, and fell within the ranges previously reported for quinoa (Supplementary Figure 1; Bertero et al., 2004; Zurita-Silva et al., 2014; Choukr-Allah et al., 2016; Lesjak and Calderini, 2017). In this study, the year of harvest was an important determinant of yield, with yields plummeting in 2018 in most cultivars. Q3 cv. and Puno cv. were less affected by environmental changes among years compared to the rest of cultivars. Regalona cv. experienced larger yield penalties in the present study than in previous works assessing drought stress (Fischer et al., 2013). Thus, besides the genetic background, other factors might be responsible for lowering yields of quinoa under field conditions, which might include high night temperatures or high temperatures combined with low precipitations or long photoperiods during sensitive phases that go from flowering to the end of grain filling stage (Bertero et al., 1999; Stikic et al., 2012; Lesjak and Calderini, 2017). Furthermore, on average, seed size showed larger values in 2018 (Figure 2B). Seed size might impact yields, and the environmental conditions seem to play an important role in controlling it. For instance, in quinoa, temperatures are shown to influence seed size during grain filling, especially in certain genotypes, which fits well with the results here presented (Bertero, 2021).

Yield can also be an important trait for breeding programs when pursuing improvements in quinoa seed quality. In fact, yield is positively correlated with fat and fiber contents and lower protein contents (De Santis et al., 2016; Curti et al., 2018). Besides, the relationship between yield and nutritional quality-related traits such as the protein content, is influenced by the genotype and the environment, in quinoa and other crops (Caballero et al., 2015; Halford et al., 2015; Curti et al., 2018). For this reason, a deeper analysis of yield influence in seed quality traits was performed (Table 4) and an important negative effect of yield on protein content was found, agreeing with the trade-off described by Curti et al. (2018) for winter sowing and with other similar findings in quinoa (De Santis et al., 2016; Präger et al., 2018; Reguera et al., 2018; Prieto et al., 2021) and other crops, including cereals (Simmonds, 1995; Rondanini et al., 2019). Nonetheless, both, yield and protein content, have also been shown to increase simultaneously when fertilizing with N (Caballero et al., 2015). Likewise, yield also correlated with most seed quality traits, but not with morphologic ones like 1000 seed weight (Table 4 and Supplementary Figure 4), indicating that seed weight is not the main contributor to yield performance (Curti et al., 2014, 2018).

Nonetheless, a correlation analysis provides a limited view of the complex interrelation that can occur between different seed traits, not showing directionality or indirect effects on other nutrient levels (Dewey and Lu, 1959). A sequential path analysis allows the classification of different variables as first-order, second-order, third-order (and so on) predictors of a response variable, so the different intercorrelations can be unraveled. Other studies have performed path analysis to study the contributions of physiological crop traits to yield performance in quinoa and other crops (Mohammadi et al., 2003; Bhargava et al., 2007; Mubai et al., 2020). However, to our knowledge, this is the first study attempting to explain quinoa seed germination through other seed characteristics. The model postulated in this work suggests that different mineral contents play an important role in determining germination rates of quinoa seeds, mainly K content, positively, and Mn and Mg contents, negatively. Different studies have linked mineral nutrition with seed germination capacity, including fertilization treatments with K increasing the germination rates of cotton (Sawan et al., 2011), or seedling growth inhibition in horse gram, after applying high concentrations of Mn (Kumari et al., 2016). However, none of these studies paid attention to the seed content of these elements. Noteworthy, it is important to highlight that the path model here presented is designed within the context of a particular environment and may work differently in other areas of cultivation. For instance, when growing quinoa in high salinity soil, the antioxidant contents may play a major role (not a secondary one) in germination capacity, where a lower germination rate may be achieved in seeds that accumulate K and Mg (Koyro and Eisa, 2008; Panuccio et al., 2014). Besides, environmental factors such as temperature, photoperiod lengths, and precipitations (throughout the crop life cycle) could influence germination rates and seed viability. Thus, the lower germination rates and seed viability observed in 2018 could be related to the shorter photoperiods after anthesis compared to 2017 and 2019 and/or with the lower temperatures at seed filling stage. Nonetheless, the specific role of temperatures, photoperiod lengths, and/or high precipitations regulating germination and viability should be further analyzed.

Overall, differences depending on genotype and on the environmental factors together with the genotype x environment interaction were found in most parameters measured (Supplementary Table 3). The cultivars analyzed had different genetic backgrounds and were grown under different environmental contexts. These variations can be seen in Figure 7, where variables have been reduced to principal components through a PCA. The factor that made the difference between seeds was the year of cultivation, as Figure 7 shows, being 2019 seeds low on component 1, 2017 seeds high on component 2, and 2018 seeds low on component 2 and high on component 1. This means that 2018 seeds show low yields and antioxidants, but high protein, amino acids, P, Cu, Mn, and Zn contents, while 2019 seeds have lower amino acids contents but higher yields and germination rates.

In 2018, the sowing date was delayed because of heavy rainfalls, so in the first two months of development, plants were exposed to higher temperatures than those sown in 2017 or 2019 (Supplementary Table 1 and Figure 1). All three growing seasons showed long photoperiods, which peaked in June at 15 h long days. Plants in 2017, 2018, and 2019 were sown with daylengths of 13 h, 15 h, and 14 h, respectively; they reached the highest photoperiod in their second month, first week, and first month, and were harvested at daylengths of 13 h, 10.75 h, and 12.25 h, respectively (Figure 1). Quinoa is a facultative short-day plant where photoperiods longer than 12 h can disrupt seed filling and maturation, although day-length neutral varieties have been developed in order to introduce the crop to higher latitudes (Bendevis et al., 2014; Zurita-Silva et al., 2014). The combination of long photoperiods and high temperatures can cause yield penalties in quinoa, especially during flowering (Bertero et al., 1999; Bertero, 2003; Lesjak and Calderini, 2017). The combination of these factors was observed in 2018 (Supplementary Table 1 and Figure 1) which could explain the poorer yield performance. Furthermore, according to the path model presented in this work, lower yields would have caused higher protein contents in seeds (Figure 5) but, ultimately, lower germinations (Figure 3).

It is important to point out that an important nutritional seed trait such as the protein content negatively correlated with yield, which is an important criterion for breeding programs (Zurita-Silva et al., 2014), and with the antioxidant capacity, which is another very interesting trait for human nutrition due to the accompanying health benefits (Figure 7 and Supplementary Figure 4; Vega-Gálvez et al., 2010). Thus, from an agronomical point of view, Puno cv. could be the best choice for cultivation, since it was the cultivar that better performed in 2018 in comparison with the rest of cultivars. However, that would have meant having smaller seeds with low values for most of the nutritional parameters. Therefore, from a nutritional perspective, a more appropriate cultivar for this area of study would be Titicaca cv., which showed higher protein and amino acids contents, especially in 2018, and also a greater antioxidant capacity.

These differences between cultivars and their performance according to the environment, together with the trade-offs among important crop characteristics, pose a challenge for breeders, who will need to study closely different cultivars for each location of cultivation. Therefore, the information presented in this work will greatly help the efforts of quinoa establishment in Northwestern Spain. This also highlights the need for in-depth research to unravel the mechanisms that cause the variations observed in the nutritional traits due to changes in the environmental conditions and agroecological contexts, with the ultimate goal of obtaining better adapted and more nutritious seeds toward contributing to food security worldwide.

## Conclusion

The results here presented highlight a great influence of the environmental conditions on the nutritional and physiological characteristics of quinoa seeds, which affects overall seed quality. Particularly, this work has shown the important effect of the environment on the amino acid balance and content, the impact of the genotype on the antioxidant capacity, protein amount, the negative correlation between protein and antioxidant contents and the existence of stable nutritional components such as the Na content. Indeed, plants grown during the second year (2018) showed lower yields and heavier seeds presenting worse germination powers. These seeds also presented higher amino acids, phosphorous, copper, manganese, and zinc contents, and lower potassium and antioxidants. According to the analyses performed, yields were associated with seed viability and protein, phenol, K, and Zn contents. Furthermore, germination rates were found to be directly influenced by K, Mg, and Mn seed contents. It is expected that these findings will help to maximize quinoa productivity and/or nutritional quality, especially for comparable climatic areas of analysis. Moreover, although the main goal of this study was to evaluate the impact of the environment on quality-related traits, it also highlights that there are still important limitations in the agronomical adaption of quinoa to these areas of cultivation which are characterized by having intense precipitations at early growth stages and high temperatures at later stages of the crop development. In line with this, we found that agronomically, varieties such as Puno cv. or Q3 cv. might be better adapted to these conditions, but Titicaca cv. and Vikinga cv. showed better nutritional properties, as they possessed higher protein contents. Altogether, this study supports the huge potential of this crop by choosing the appropriate variety according to the area of interest. Therefore, the selection of the cultivar must be well informed, paying careful attention to how the seeds respond to the climatological characteristics in a particular location.

## Data Availability Statement

The original contributions presented in the study are included in the article/Supplementary Material, further inquiries can be directed to the corresponding author/s.

## Author Contributions

MR, NA, LFP-R, NF-G, and JM conceived and planned the experiments. SG-R, LFP-R, IG, JM, NA JP, and CH carried out the experiments. SG-R, MR, IM, JM, NA, JP, CH, NF-G, and LB contributed to the interpretation of the results. MR and SG-R took the lead in writing the manuscript. All authors provided critical feedback and helped shape the research, analysis, and manuscript.

## Conflict of Interest

The authors declare that the research was conducted in the absence of any commercial or financial relationships that could be construed as a potential conflict of interest.

## Footnotes

1 http://rsbweb.nih.gov/ij/
