# Supplementary material for: Studying the Impact of Different Field Environmental Conditions on Seed Quality of Quinoa: The Case of Three Different Years Changing Seed Nutritional Traits in Southern Europe
Source: Front Plant Sci. 2021 May 12;12:649132. doi: 10.3389/fpls.2021.649132 (PMC8149766; doi:10.3389/fpls.2021.649132)
Supplement: Supplementary file 1 [file Data_Sheet_1.pdf]

Supplementary Table 1. Climatological parameters. Monthly average temperature and water supply in the experimental field of Zamadueñas (Spain) during the growing seasons of 2017, 2018 and 2019.

**Year**		**Month**	**Minimum temperature (°C)**	**Maximum temperature (°C)**	**Mean temperature (°C)**	**Total precipitations (mm)**	**Total irrigation (l/m²)**	**Total water received**
**2017**	**05/04/2017**	April	3.83	21.07	12.58	3.80	81.00	84.80
		May	9.02	24.49	16.69	42.00	102.00	144.00
		June	13.69	31.35	22.43	5.40	102.00	107.40
		July	13.47	31.02	22.43	33.20	48.00	81.20
		August	13.35	30.64	21.84	13.60	-	13.60
	**total**					98.00	333.00	431.00
**2018**	**18/06/2018**	June	12.09	25.20	18.24	70.60	30.90	101.50
		July	13.10	31.46	21.95	22.20	98.40	120.60
		August	13.61	31.88	22.48	0.00	108.00	108.00
		September	12.60	29.18	20.33	28.00	39.00	67.00
		October	5.89	19.75	12.11	19.81	-	19.81
	**total**					140.61	276.30	416.91
**2019**	**03/05/2019**	May	6.00	22.64	14.40	7.00	62.50	69.50
		June	10.13	28.03	19.28	6.00	63.96	69.96
		July	14.64	31.83	22.88	29.80	72.00	101.80
		August	13.54	30.39	21.80	21.80	18.00	39.80
		September	10.85	25.37	17.47	23.60	-	23.60
	**total**					88.20	216.46	304.66

Supplementary Table 2. Soil analysis. A soil characterization was performed each year (2017, 2018, and 2019) in the cultivation area.

	**Clay %**	**Sand %**	**Silt %**	**Soil texture**	**pH in water (1:5)**		**Soil electrical conductivity (EC) µS/cm**		**K mg K_2O/g**		**Organic mater %**		**P mg P_2O_5/Kg**		**N %**	
**Year**					**Mean**	**Stand Dev**	**Mean**	**Stand Dev**	**Mean**	**Stand Dev**	**Mean**	**Stand Dev**	**Mean**	**Stand Dev**	**Mean**	**Stand Dev**
2017	15	47	38	clay-silty-loam	8.58	0.29	0.083	0.036	0.334	0.04	2.03	0.26	48.04	5	0.075	0.015
2018	18	38	43	clay-silty-loam	8.15	0.29	0.053	0.013	0.419	0.05	1.19	0.18	105.07	11	0.068	0.014
2019	18	38	44	clay-silty-loam	8.55	0.29	0.067	0.013	0.472	0.06	1.28	0.19	85.47	9	0.067	0.013

Supplementary Table 3. Two-way ANOVA for different quinoa seed qualitative traits from 6 cultivars harvested in three years.

Variable	Year (F value)	Cultivar (F value)	Year x Cultivar interaction (F value)
Seed Weight	15.966**	13.822**	2.281*
Seed Area	20.608**	56.071**	11.020**
Germination rate	118.105**	10.358**	13.942**
Viable seed rate	353.223**	13.499**	16.924**
Saponin content	20.845**	27.430**	3.392^{ns}
Protein Content	44.559**	6.710**	2.146*
P content	83.658**	21.678**	13.262**
K content	143.974**	10.314**	2.608*
Ca content	4.899*	3.069*	3.193**
Mg content	5.779**	5.471**	1.929^{ns}
Na content	0.892^{ns}	2.332^{ns}	1.522^{ns}
Fe content	3.510*	5.388**	2.063^{ns}
Cu content	5.201*	5.205**	2.191*
Mn content	172.248**	82.810**	36.096**
Zn content	112.663**	3.758**	1.075^{ns}
FRAP value	40.289**	26.428**	2.846*
Total phenolic content	38.472**	8.566**	1.062^{ns}
Total flavonoid content	39.505**	13.238**	3.737**

ns: non significant; ** significant at 99% confidence; *significant at 95% confidence

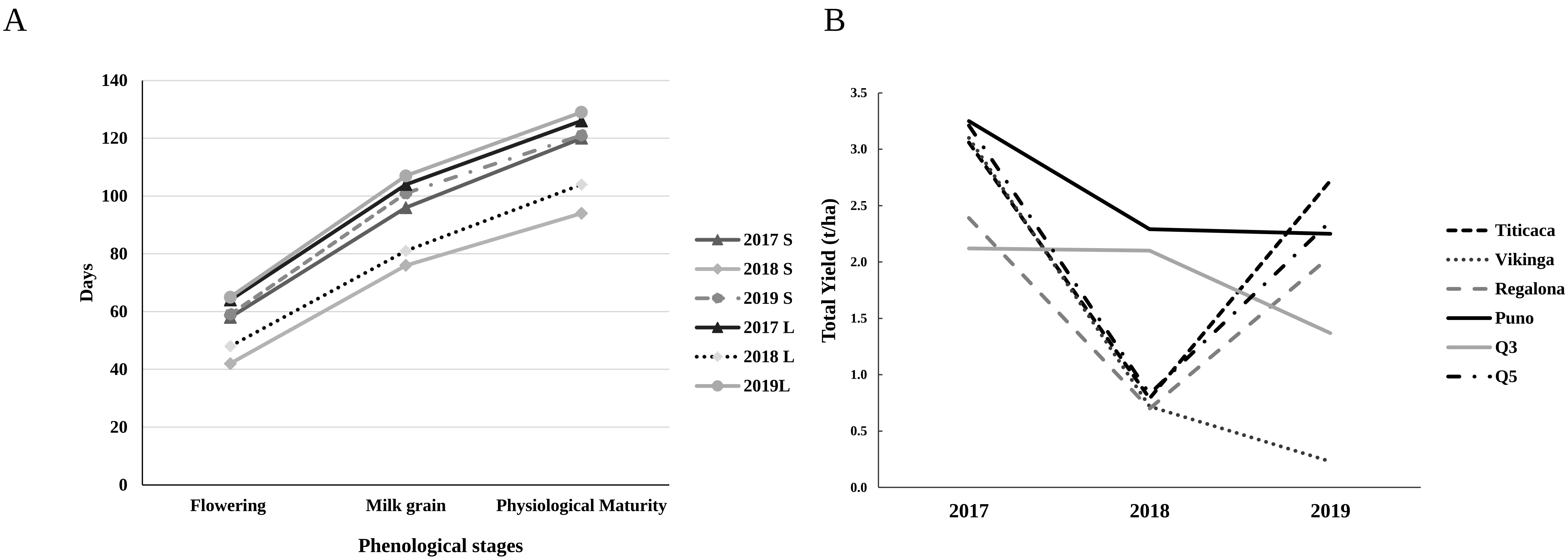

Supplementary Figure 1. Life span and total seed yield (t/ha) among years and quinoa varieties. Sowing in 2017 took place on April 5th and harvesting on August 8th, in 2018 sowing date was June 18th and harvesting on October 25th and in 2019 sowing took place on May 3rd and harvesting on September 20th. A) According to the life span of the different varieties, two groups can be distinguished: those with longer life cycles (Vikinga and Q3; L) and those with shorter cycles (Q5, Puno, Titicaca and Regalona; S) B) Total seed yield was determined each **year** for each variety and is expressed as t/ ha.

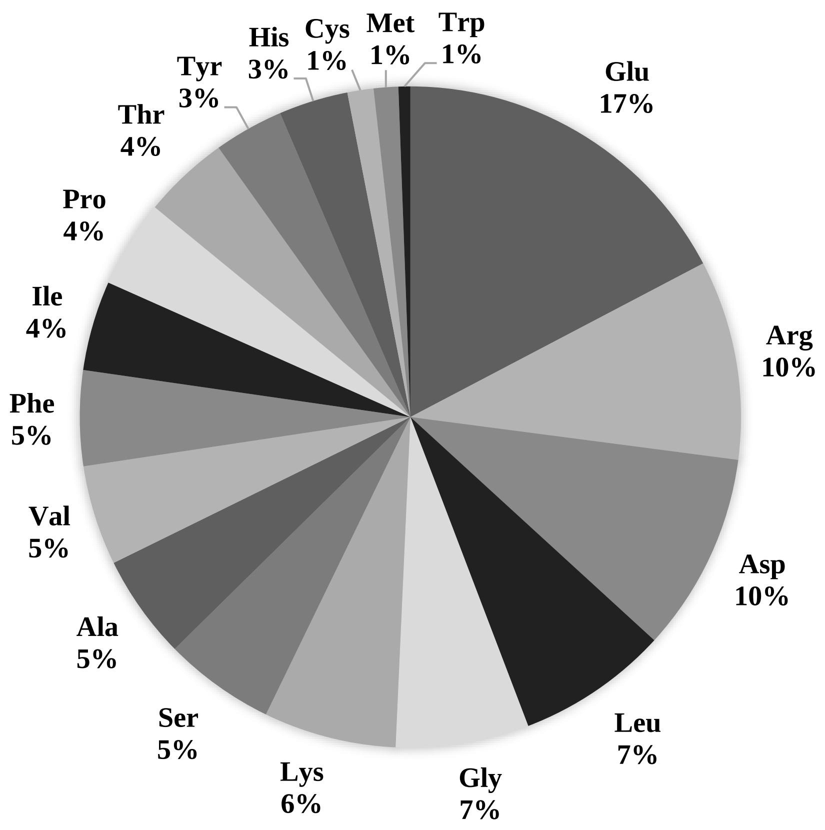

Supplementary Figure 2. Amino acid profile as mean values of amino acids in all cultivars and years and presented as percentage of crude protein.

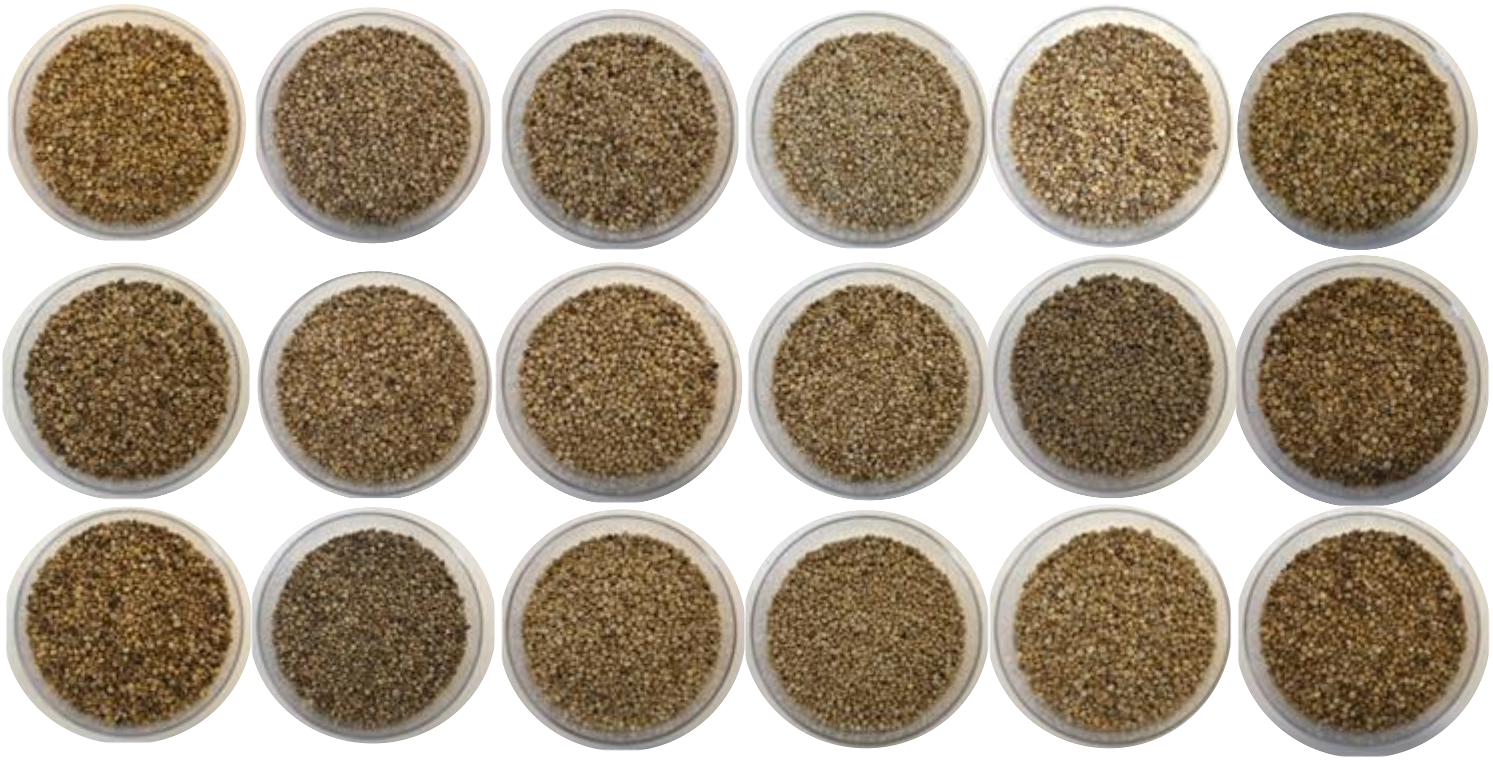

Supplementary Figure 3. Pictures of seeds used for color parameter analysis.

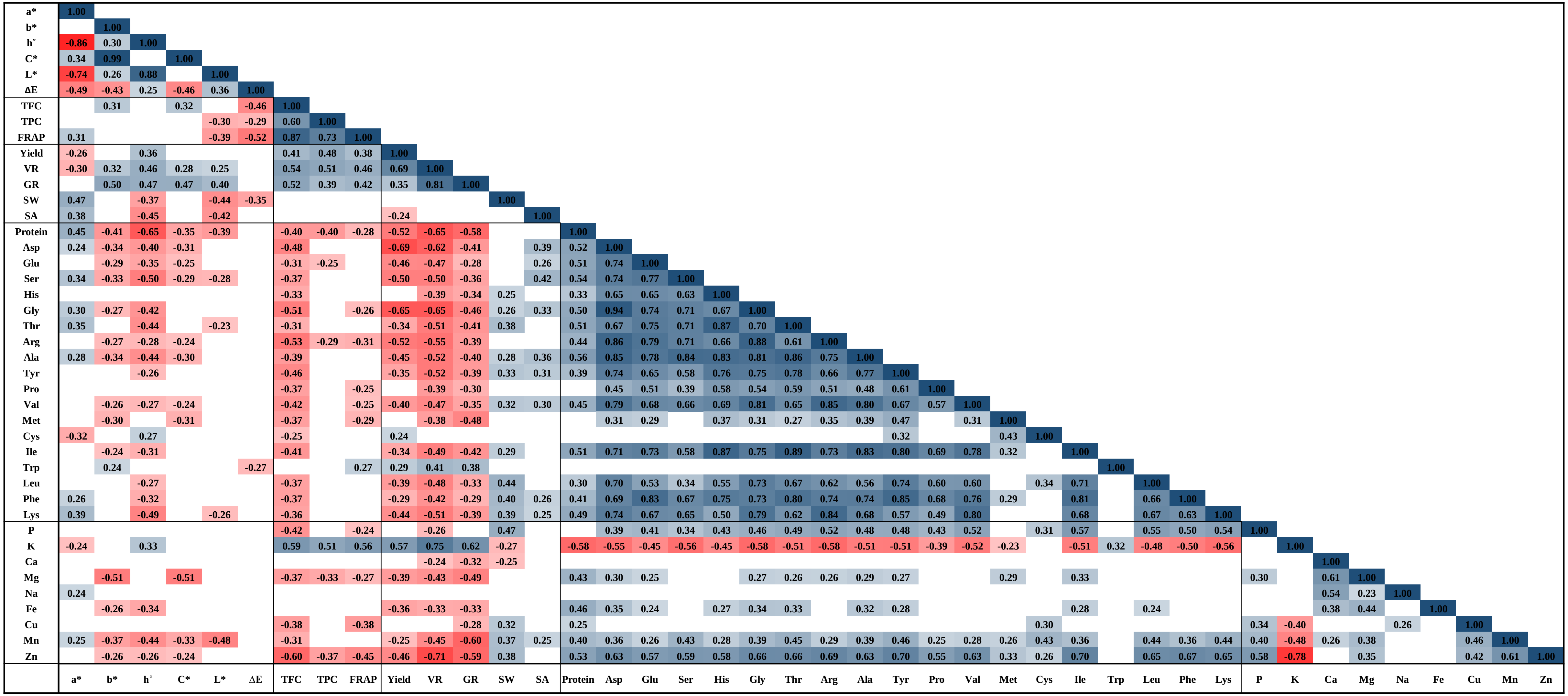

Supplementary Figure 4. Correlogram of variables measured. Pearson correlation coefficients (r) are given when the correlation between variables is statistically significant (*p*<0,05). Red cells indicate negative correlations and blue cells show positive correlations. TFC: total flavonoid content, TPC: total phenolic content, VR: viability rate, GR: germination rate, SW: seed weight, SA: seed area.
